# Metabolomic Profiling of *Endomyces magnusii* During Long-Term Cultivation on Glycerol and Glucose

**DOI:** 10.3390/jof12080592

**Published:** 2026-08-10

**Authors:** Olga I. Klein, Katerina V. Sazanova, Elena P. Isakova, Natalya N. Gessler, Alexander M. Prosvirin, Ekaterina V. Solovyeva, Yulia I. Deryabina

**Affiliations:** 1 A.N. Bach Institute of Biochemistry, Research Center of Biotechnology of the Russian Academy of Sciences, Leninsky Ave. 33/2, 119071 Moscow, Russia; klein_olga@list.ru (O.I.K.); elen_iss@mail.ru (E.P.I.); gessler51@mail.ru (N.N.G.); prosvirin1811@mail.ru (A.M.P.); 2 Komarov Botanical Institute of the Russian Academy of Sciences, st. Professora Popova, 2, 197022 St. Petersburg, Russia; ksazanova@binran.ru (K.V.S.); kat535441@yandex.ru (E.V.S.); 3 Archive of the Russian Academy of Sciences, St. Petersburg Branch, Kievskaya Street 5, 196084 St. Petersburg, Russia

**Keywords:** yeast, aging, metabolomic profile, metabolites, carbon sources

## Abstract

Introduction: The study purpose was to identify possible key metabolites that determine the adaptation of the Endomyces magnusii yeast to long-term cultivation (four weeks) using glycerol as an “oxidative” and glucose as a “fermentative” substrate. Methods: The metabolic profile was assayed using gas chromatography combined with mass spectrometry, followed by bioinformatic analysis (PARADISe, Golm metabolome database (GMD), MassBank, UniChrom). Results: PCA and PLS-DA analyses showed that the type of carbon source contributed significantly to the overall variability of the data, and the greatest variance was observed for the groups grown on different substrates for the first cultivation week. Growth on glycerol increased the chronological lifespan of E. magnusii due to the early launch of adaptive oxidative stress, the active use of lipids as an energy source, the accumulation of membrane sterols, osmo-protective polyols, organic acids (malic, methyl glycerinic, palmitic, linoleic), and some sugars (lyxose, galactose), which increased the overall resistance and maintained high cell survival. On the contrary, cultivation using glucose provoked a sharp substrate depletion, inducing passive storage of sugars (trehalose), diauxic shock, and less effective antioxidant protection, which provided lower cell survival upon prolonged growth. Conclusions: (1) Metabolic signs associated with prolonged culturing were identified in all the compounds classes tested (polyols, fatty acids, lactones); (2) some metabolites (in particular, dulcitol), being hypothetical biomarkers of aging, are at the same time protective agents involved in the adaptation of yeast cells to the deep stationary growth stages. Our data can serve as a basis for comparative studies of aging-related metabolism in other eukaryotic models.

## 1. Introduction

Aging is a natural biological phenomenon showing a gradual and progressive decrease in physiological functions at all levels, namely molecular, organelle, cellular, tissue, and organism, that increases the risk of various pathologies and death of the organism [1,2,3]. Accumulation of irreversible damage to macromolecules due to excessive generation of free radicals, some spontaneous errors in biochemical reactions, and biologically programmed decrease in functions are suggested as the main reasons for the aging process [4,5,6,7,8].

The study of the aging molecular mechanisms, either to reduce or eliminate the associated symptoms, dictates the need for close interaction of basic postgenomic “omics” sciences, namely transcriptomics, proteomics, and metabolomics, which can provide some additional information about the changes in the organism at “postgenomic” molecular levels (from transcripts to low molecular weight substances) [9]. However, metabolomics plays a special role in gerontological research, since the metabolome, being the end of all biological events in the body, can provide information about all the molecular mechanisms of aging. Metabolomics is the latest technology in the “omics” sciences; it focuses on obtaining an integral image of the current metabolic status of an organism via a diverse set of metabolites related to some physiological and pathophysiological processes [10]. A lot of metabolites, both endogenous and exogenous, which are the end products of gene expression and protein regulation, can be assayed using metabolomic instruments, identifying various physiological and/or pathological conditions and reflecting the complexity of the aging process [11]. Biomarkers of aging can also predict the risk of future age-related morbidity and mortality. As a precondition for identifying the biomarkers of aging and long-life expectancy, numerous studies have attempted to learn how the metabolome and proteome change with age, both in humans and in model systems, including worms [12,13,14], fruit flies [15,16,17,18], and mice [19,20,21,22]. Altogether, the published results showed that both proteomic and metabolomic technologies can detect biomarkers of age and related diseases. The lower eukaryotes, namely yeast, are also an excellent model for studying cell and organism aging at the level of single cells [23,24,25,26,27,28].

The studies using budding yeast of *Saccharomyces cerevisiae* as a model organism have provided a deep understanding of the molecular mechanisms of cell and organism aging in multicellular eukaryotes and showed that the main features of biological aging are evolutionarily conserved [23].

Moreover, their short lifespan and compatibility with high-tech methods and approaches are essential advantages for using yeast as a single-cell model to study various metabolic processes. The aging of *S. cerevisiae*, the baker’s yeast widely used in research, is studied on two main models, namely replicative and chronological models [25,29]. Replicative lifespan is defined as the number of the mother cell’s divisions until replicative aging. The study of replicative aging allows us to model aging mitotic cells of multicellular organisms [30]. Chronological lifespan refers to the amount of time the yeast survives in the stationary growth phase after nutrient depletion [31]. The type of aging is caused by the inhibition of yeast growth reaching a critical state, usually due to depletion of essential nutrients and accumulation of toxic metabolites in the medium. Therefore, the chronological and replicative (based on the budding potential) lifespan of *S. cerevisiae* is a crucial factor in the studies of aging. Titorenko’s group’s research has shown that different inter-compartment communications (i.e., organelle–organelle and organelle–cytosol interactions) within a yeast cell play a significant role in the chronological aging of yeast [32,33].

Previously, we studied the metabolic profile of the obligately aerobic yeast of *Endomyces magnusii* with a complete respiratory chain and a well-developed mitochondria system using glycerol and glucose as carbon sources [34]. The *E. magnusii* yeast is an obligate aerobe with a complete respiratory system similar to the mitochondria system of higher animals, which makes them a unique model for studying some mechanisms, including aging. The membrane and mitochondria apparatus of the yeast with a fermentative type of metabolism, namely *S. cerevisiae*, is poorly developed, with only a few large mitochondria with small cristae in the cell. Its mitochondria, being irregular in their shape, structure, and packaging, have fewer cristae. However, the yeast with a pronounced aerobic metabolism, namely *E. magnusii*, has a well-developed membrane apparatus with abundant mitochondria with numerous cristae. The mitochondrial respiratory chain in traditional yeast species, such as *S. cerevisiae*, differs from the complete respiratory chain, in particular, the absence of complex I. The *E. magnusii* yeast has complete complex I and has no alternative respiratory pathways [35]. Thus, this type of yeast should be an excellent alternative to the classical object for the research, namely baker’s yeast, and serve as a model more similar to higher eukaryotes.

In our previous study [34], we assayed the activity of antioxidant systems, namely catalases (CATs) and superoxide dismutases (SODs), enzymes of the glutathione system (glutathione peroxidase and reductase), aconitase, and the key enzymes maintaining the NADPH level in the cells (glucose-6-phosphate dehydrogenase and NADP^+^-isocitrate dehydrogenase) upon yeast cultivation using two types of substrates. Also, the antioxidant status (the dynamics of oxidized and reduced glutathione, diene conjugates, and ROS) in the cells at different growth stages, including the deep stationary stage, has been assessed. Our study revealed a much higher level of ROS, increased activity of antioxidant systems (CATs, SODs, and the enzymes of the glutathione system), and a high redox potential of the culture in both the late and deep stationary growth phases upon glycerol utilization. Moreover, we observed a high survival rate of the yeast population using glycerol, while in glucose-assimilating cultures, it decreased significantly by the seventh day of growth. We believe that this phenomenon may reflect some symptoms of yeast aging, which in the early stages (the first week of cultivation) is accompanied by active adaptation of the ROS-induced antioxidant systems, stimulation of NADPH, and reduced glutathione synthesis. It, in turn, maintains the redox potential of yeast cells in the early stages of aging. The data confirmed the hypothesis that the ROS formed upon oxidative phosphorylation in the eukaryotic mitochondria at all levels of life organization, from yeast to humans, playing a crucial role in regulating life span [36]. According to the hypothesis, the intermediates of the Krebs cycle, namely α-ketoglutarate, and the mitochondria ROS regulate the activity of transcription factors, for example, Hif1, which leads to a significant restructuring of cell metabolism. The key metabolic pathways also affect the ROS levels and antioxidant signaling, thereby mutually regulating lifespan.

Subsequently, while studying the role of calorie restriction in regulating chronological aging [37], we demonstrated that in spite of 90% population survival in all the variants of cultivation on both glucose and glycerol, the ability to form colonies and the cell metabolic activity significantly decreased upon growth. We also confirmed that in the *E. magnusii* population, there are indeed some aging processes. The results confirmed our assumption that in the glycerol-assimilating cells of *E. magnusii*, i.e., under the conditions of calorie restriction, H_2_O_2_ generation at the stationary growth stage increases, followed by the rise in the SODs activity that prolongs the chronological lifespan of the population. Then, we found that autophagic processes in some fractions of the cell population, maintaining a high level of lipid components in the late stationary stages of cultivation upon substrate depletion and calorie restriction, could provide the increase in the chronological lifespan of *E. magnusii* [38].

The study purpose is to assay the metabolic profile of the *E. magnusii* yeast upon long-term cultivation using two types of substrates, namely glycerol as an “oxidative” type of substrate, and glucose as an “fermentative” type of substrate, to search for possible key marker metabolites involved in the adaptation of the yeast to the aging process and to identify possible metabolic mechanisms ensuring the population survival upon long-term cultivation.

## 2. Materials and Methods

### 2.1. Yeast Strain and Culture Conditions

The *E. magnusii* yeast was raised in batches of 100 mL either in glycerol (1%)- or glucose (1%)-containing medium at 28 °C. The composition of the medium contained (g/L): CaCl_2_—0.05, MgSO_4_—0.5, KH_2_PO_4_—8.6, NaCl—0.1, (NH_4_)_2_SO_4_—0.3, the yeast extract—2.0, *L*-tryptophan—2.75 mg, *L*-methionine—2.75 mg, and *L*-histidine—2.75 mg at 28–29 °C as described before [39]. The cell suspension was assessed using absorbance at the wavelength of 590 nm (A_590_) with a Specol-11 spectrophotometer (Carl Zeiss, Oberkochen, Germany). Cells were harvested in the different growth phases, namely the late stationary (1 week of growth, A_590_ = 4.4–4.7), deep stationary 1 (2 weeks of growth, A_590_ = 4.4–4.7), deep stationary 2 (3 weeks of growth, A_590_ = 4.4–4.7), and deep stationary 3 (4 weeks of growth, A_590_ = 4.4–4.7) stages.

### 2.2. Sample Preparation and Extraction

The *E. magnusii* culture was raised using centrifugation at 15,000× *g* for 15 min, washed twice with distilled water, and then pressed on filter paper. Samples were prepared according to Oliver Fink’s method [40,41] with some modifications [42]. A 200 mg sample was immersed in ice-cold methanol–chloroform–water (2 mL) to quench metabolism. The samples were then homogenized and dissolved in cold methanol–chloroform–water. The mixture was shaken for about 0.5 h and kept at +4 °C for 12 h, then shaken again for about 0.5 h and centrifuged at 15,000× *g* for 15 min. After extraction, the samples were cleaned of cell debris by centrifuging for 10 min at 15,000× *g* at 4 °C. The resulting extract was treated in a vacuum evaporator. The dried sediment in pyridine (30 µL) was applied to the internal standard (tricosane, normal hydrocarbon of C23) and BSTFA (N,O-bis—3-methyl-silyl-3-F-acetamide, 30 µL). The mixture was incubated at 90 °C for 20 min. The amount of low molecular weight organic compounds in biofouling was assayed by gas chromatography—mass spectrometry (GC-MS). The experiment was performed in three biological replicates (for each substrate (glycerol, glucose), at every growth stage). The total number of samples for each substrate was 15 (3 samples for each growth stage). To control the reproducibility of the GC-MS analysis, one of twenty-four samples (selectively) was assessed once more to ensure that the obtained data were reproducible.

### 2.3. Gas Chromatography Coupled with Mass Spectrometry

Samples were analyzed using gas chromatography together with mass spectrometry using a gas chromatograph Maestro instrument (Interlab, Moscow, Russia). Separation was performed using a HP-5MS, 30 m × 0.25 mm × 0.25 µm column with a carrier gas of helium, a constant flow of 1 mL/min. Chromatography was performed with linear temperature programming from 70 °C to 320 °C at 6 °C/min. The peaks were recorded using an Agilent 5975 mass-selective detector (Santa Clara, CA, USA). Mass spectra were scanned in the range of 50–750 *m*/*z* with a frequency of 1.6 scans/sec. Chromatograms of the samples were recorded using the total ion current. To ensure the reproducibility of the chromatography, a mixture containing 5 µL of each sample was assayed at the beginning and end of the sample sequence.

### 2.4. Identification of Metabolites and Semi-Quantitative Analysis

PARADISe performed PARAFAC2 deconvolution simultaneously across all samples, without using an internal alkane ladder or a separate calibration file, according to the PARADISe tutorial [43].

- CDF files of all samples are loaded together.
- The user manually selects intervals corresponding to chromatographic peaks.
- For each interval, a PARAFAC2 model is fitted to all samples simultaneously, decomposing the three-way array (samples × scans × m/z) into components.
- The model resolves the elution profile, the purified mass spectrum, and the relative concentration (score) in each sample for every component.
- The final Peak Area table contains peak areas calculated as the product of the elution profile and the score for each sample.

PARADISe does not perform absolute quantification—the output consists of peak areas. The internal standard C23 is present in the chromatogram as a regular peak and is not used by the software for concentration calculations.

Retention Index (RI) calculation.

Retention indices (RI) were calculated from the retention times (rt) reported by PARADISe using a 4th-order polynomial approximation:

RI = −0.0005 × rt^4^ + 0.052 × rt^3^ − 0.9283 × rt^2^ + 47.42 × rt + 793.94

The coefficients were obtained empirically by least-squares fitting to the measured retention times of n-alkanes (C10–C40) chromatographed on the same column type (HP-5MS or VF-5MS, 5% phenyl/95% dimethylsiloxane) under identical instrumental conditions. The reproducibility of RI values is within 5–15 index units, which is sufficient for the supporting role of RI in metabolite identification (Level 2 requires agreement with NIST WebBook RI within ≤20 units for 5–phenyl columns, in addition to mass spectral match MF > 800). For identification, two orthogonal criteria were used.

Criterion 1—Match Factor (MF). Cosine similarity between the measured and library mass spectra (scale 0–1000). It accounts for matching *m*/*z* values and intensities but does not incorporate structural information (e.g., expected fragment patterns for a compound class). MF > 800 was considered a necessary but not sufficient condition for identification.

Criterion 2—RI. The measured RI was compared with NIST WebBook values for columns of the same stationary phase type (HP-5MS, HP-5, VF-5MS, DB-5MS—5% phenyl/95% dimethylsiloxane; SE-30, OV-1, SPB-1—100% dimethylsiloxane). For 5–phenyl columns, a tolerance of ≤20 RI units was applied; for 100% dimethylsiloxane columns, ≤40 RI units, accounting for the known systematic offset between these phases.

We also used the library of mass spectra provided by the resource center “Development of Molecular and Cellular Technologies” of St. Petersburg State University and the library of the Laboratory of Analytical Phytochemistry of the Botanical Institute of the Russian Academy of Sciences (St. Petersburg, Russia).

### 2.5. Potential-Dependent Staining

Potential-dependent staining of mitochondria was performed in the *E. magnusii* cells raised in different growth phases by MitoTracker™ Red CMXRos. Cells were incubated with 0.5 µM MitoTracker™ Red CMXRos and examined at 0, 15, 20, and 30 min. The incubation medium contained 0.01 M PBS, pH 7.4; and 1% glycerol or glucose, respectively. Regions of high mitochondrial polarization are indicated by red fluorescence due to the concentrated dye. To examine the MitoTracker™ Red CMXRos -stained preparations, filters 02, 15 (Zeiss) were used (magnification ×100). The photos were taken using an AxioCam MRC camera.

### 2.6. Cell Viability and Vitality Assays by Flow Cytometry

Cells were centrifuged and resuspended in medium to determine cell concentration and viability using propidium iodide (PI). For the viability assay, PI staining and acquisition were performed as follows. Cells (1 mL) were transferred into a 15 mL conical tube, centrifuged at 3000× *g* for 10 min, and resuspended in 100 μL of PBS. After two washes in PBS, cells were resuspended in 1.5 mL PBS and divided into three aliquots 500 μL each (negative control, positive control and a sample tested). The positive control was heated up to 100 °C for 5 min. Then, 0.5 μL PI was added to the cells of the positive control and the sample, stirred, and incubated at 37 °C for 30 min. Acquisition was performed on a cytometer CytoFLEX^®^ (Beckman Coulter, Brea, CA, USA). For cells stained with PI, the maximum fluorescence excitation was used—535 nm (green laser), maximum emission—617 nm (red channel). The data obtained were analyzed using the CytExpert software (version 2.4.0.28) and visualized using the FlowJo software (version 10.8.1).

### 2.7. CLS Assay

The samples of the cultures at various growth stages were diluted to assay the total number of cells using microscopy. Serial dilutions of the yeast cultures were inoculated on solid YPD (1% yeast extract, 2% peptone, 2% dextrose, and 2% agar) medium in two replicates to count the number of live cells in each version. The cultures raised using 1-% glycerol and 1% glucose were washed and suspended in distilled sterile water, then diluted up to a final density of 106 and 107 cells mL^−1^, respectively. The plates were kept at 29 °C for 24–48 h. We calculated the number of colony-forming units (CFU) as the number of reproductively capable cells in a sample as follows: number of CFU × dilution × factor × 10 = number of reproductively capable cells per mL. The colony growth was inspected after 48 h, and the number of grown colonies was counted on each plate. The total number of cells was assayed under a light microscope using Gorjaev’s chamber (×400) on at least 1000 cells in one biological replicate.

### 2.8. Preparation of Cellular Homogenate

The cellular homogenates from the cells raised in different growth phases were obtained according to the standard protocol as described before [44]. Cells were twice washed with ice-cold water, centrifuged at 3500× *g* for 10 min, and disrupted with a mortar using liquid nitrogen for 2 min. The obtained homogenate was resuspended in grinding medium (1:5 *w*/*v*). The medium contained 20 mM KP_i_, pH 7.2; 2 mM EDTA, 0.5 mM phenyl-methylsulfonyl-fluoride (PMSF). Then the homogenate was centrifuged at 10,000× *g* for 30 min. The supernatant was used for the experiments. Cellular proteins were assayed by Bradford method [45] with BSA as standard.

### 2.9. Antioxidant Enzyme Activities Assay

Total CATs activity was assessed by monitoring the cellular homogenate at 240 nm with an SF-2000 spectrophotometer (Spectr, Sankt Peterburg, Russia). The procedure was performed in 20 mM KP_i_ buffer, pH 7.2, containing 2 mM EDTA. The reaction was triggered by the application of H_2_O_2_ (the molar extinction coefficient 46.3 M^−1^ cm^−1^). The CATs activity was measured by monitoring the decrease in H_2_O_2_ [46]. A240 was determined every 15 s for 3 min after the start of reaction. CATs activity was expressed in µmol used H_2_O_2_ × min^−1^ × mg^−1^ of protein. The activity of SODs in cell suspension was assessed by monitoring of A406 with a Specol-11 spectrophotometer (Carl Zeiss, Oberkochen, Germany) upon inhibition of quercetin autooxidation as described in [47]. The procedure was performed in 20 mM KPi buffer, pH 10.2, containing 0.8 mM TMDA, 0.1 mM EDTA, and 1.4 µM quercetin. Fifty percent inhibition of quercetin autoxidation was considered as a SOD enzymatic activity unit.

### 2.10. Assay of the Reduced and Oxidized Glutathione

The harvested yeast cells were briefly washed with deionized water, frozen, and homogenized in liquid nitrogen. Then the homogenate was transferred to a 0.1 M KP_i_ buffer, pH 8.0, in a ratio of 1:5. Proteins were sedimented using an equal volume of 5% metaphosphoric acid (Reachim, Moscow, Russia), keeping the mixture on ice for 20 min. The extracts were centrifuged for 20 min at 15,000× *g.* The oxidized and reduced glutathione were assayed in the supernatant, 10-fold diluted in 0.1 M NaP_i_ buffer. The mixture of 100 µL extract and orthophthalic aldehyde in a 3 mL quartz cuvette was incubated in the dark for 15 min. Glutathione was assessed using the emission spectrum in the wavelength range of 360–500 nm when excited by light with a wavelength of 350 nm on a Shimadzu RF 5301 PC spectrophotometer (Shimatsu, Kyoto, Japan). The reduced glutathione amount was calculated using the area of the curve of the emission spectrum. The oxidized glutathione was performed similarly but diluted in 0.1 M NaOH. To assay the reduced and oxidized glutathione amount in each series of experiments, calibration curves with a concentration range of 1–10 µg/mL were designed using the reduced glutathione (Serva Electrophoresis GmbH, Heidelberg, Germany) and the oxidized glutathione (Diagon Reagents, Diagon, Hungary). The data were calculated per g of dry biomass.

### 2.11. Detection of the Reactive Oxygen Species ROS

The dynamics of intracellular ROS production was monitored using the spectroscopic fluorescence probe of dihydro-2’,7’-dichlorofluorescein diacetate ester (H_2_DCF-DA) (Sigma, Sant Louis, MO, USA). Upon crossing the cell membrane, H_2_DCF-DA is hydrolyzed to a fluorescence species that remains trapped intracellularly, like its oxidation product. A stock solution of 8 mM H_2_DCF-DA in dimethyl sulfoxide (DMSO) was added to a cellular suspension to final concentrations of 40 µM H_2_DCFDA and 0.1% DMSO. The suspension was incubated in PBS in the dark for 30 min in medium containing 1.2 M sorbitol, 10 mM HEPES buffer, 0.5% BSA, pH 7.4. The cells were then rinsed in the same medium, and after 5–7 min, the intensity of 2’,7’-dichlorofluorescein (DCF)-induced fluorescence was detected every 10 min for 2 h using dual wavelength photometry at 485 (λ_ex_)/528 (λ_em_) nm with a Synergy 2 (Bio Teck, Winooski, VT, USA) microplate photofluoriometer at a relative sensitivity of 100.

### 2.12. Carbohydrate Analysis

The carbohydrates were extracted with extremely hot water for 20 min four times. From the resulting extract, we removed the proteins and then purified of charged substances using a column with both the Dowex-1 (acetate form) and Dowex 50 W (H^+^) ion exchange resins [48]. The carbohydrate composition was assayed using gas–liquid chromatography with trimethylsilyl derivatives, which was obtained from the lyophilized extract [49]. We used *α*-Methyl-*D*-mannoside (“Merck”, Rahway, NJ, USA) as the internal standard. Gas chromatography was performed with a Kristall 5000.1 gas chromatograph (“Chromatek”, Yoshkar-Ola, Russia) equipped with a ZB-5 30 × 0.32 mm capillary column (“Phenomenex”, Torrance, CA, USA). The temperature program was set at +130, 5–6 °C/min gradient to +270 °C. We used the markers of glucose, mannitol, arabitol, inositol, and trehalose (“Sigma”, Sant Louis, MO, USA) as the standards.

### 2.13. Statistical Analysis

#### 2.13.1. Statistical Analysis of Metabolomic Data

To improve the quality of the analysis and ensure the correct operation of the models, the following methods were used:

- Median normalization (by row). Each feature (row) was scaled relative to its median to reduce the influence of the overall signal level.
- Logarithmization. The log(1 + x) transformation was used to reduce the influence of large outliers and bring the feature distribution closer to normal.

To visually assess the data structure and possible separation of classes, dimensionality reduction methods were used: principal component analysis (PCA), partial least squares discriminant analysis (PLS-DA), and orthogonal partial least squares discriminant analysis (OPLS-DA). The methods allow data to be projected from the original high-dimensional space into a lower-dimensional space while preserving as much information as possible about the relative positions of objects. Principal component analysis (PCA) determines the directions of greatest data dispersion and projects data along these directions, without taking into account information about target labels. PCA algorithm provides the means to achieve objective dimensionality reduction, and its application reveals group structure only when within-group variation is significantly less than between-group variation. PLS-DA is a method similar to PCA, but based on a combined decomposition of two matrices. The first matrix contains experimental measurements, and the second matrix includes a priori data, i.e., class belonging to a sample [50]. As a result, we get a matrix of scores, where objects are presented in a space of a lower dimension, but the differences between classes are maximum. OPLS is an extension of the PLS supervised regression method that includes an integrated OSC (Orthogonal Signal Correction) filter, which is most preferred for comparing two samples because OPLS-DA uses only one predictive component for binary classification. This simplifies interpretation, as all predictive power is concentrated on a single axis. The risk of overfitting is minimal [51].

PCA, PLS-DA, OPLS-DA, as well as heat map generation, were performed using MetaboAnalyst software (5.0 version). MetaboAnalyst 5.0 is available online: https://www.metaboanalyst.ca (accessed on 10 November 2025) [52]. The algorithm leave-one-out cross-validation, which is suitable for small samples, was used. The model is trained on almost the entire data set. Testing is always performed on a single independent sample that was not used in training. The process is repeated (each sample serves as a test sample). The results are stable and do not depend on random sample splitting.

#### 2.13.2. Statistical Analysis of Physiological and Biochemical Data

The experiments were performed in biological triplicate with a standard error of less than 5%. Data were estimated using one-way ANOVA (n = 3) with R (R Core Team 2016). Student’s *t*-test was used to analyze the significance of differences for independent samples. Values were considered significant at *p* < 0.05.

## 3. Results

Upon glucose limitation during the transition from the logarithmic growth stage to the stationary stage, the cells switch from fermentative metabolism (glucose-to-ethanol) to mitochondrial oxidative metabolism (ethanol and organic acids formed during cultivation). The “diauxic shift” is accompanied by some dramatic changes in transcription, translation, and metabolism, slowing cell growth using non-fermentable carbon sources [53,54]. It eventually promotes the exit from the cell cycle and a state of rest. The chronological lifespan mainly depends on the yeast’s response to substrate depletion, so the yeast CLS is considered a model of aging of either dormant stem cells or postmitotic cells [55,56].

In the presented study, we investigated the dynamics of the metabolic profiling, which reflects the stages of chronological aging in the cells assimilating a “respiratory” substrate from the very beginning of the cultivation, in addition to the classical diauxic shift in the yeast cells at the transition to the stationary growth stage upon glucose utilization.

### 3.1. Changes in Morphometric Parameters, Metabolic Activity, and Viability of the E. magnusii Yeast Cells upon Long Cultivation Using Various Substrates

The *Endomyces* (*Dypodascus*) *magnusii* yeast (Ludwig) (Division Ascomycota, class *Saccharomycotina*, order *Saccharomycetales*, family *Endomycetaceae*) was first described by Ludwig (1886) and Briffield (1891). In the asexual stage, *E. magnusii* yeast usually forms small, smooth, cream-colored colonies with short hyphae. The peripheral part of the colony is somewhat submerged, glassy, and smooth, often with a fringed, indented edge. Single cells usually bud to form mycelial and pseudomycial forms. Yeast of *E. magnusii* belongs to multinucleate fungi with aerobic metabolism and a well-developed membrane apparatus and an abundant complex of mitochondria with numerous cristae [37].

**Figure 1:**
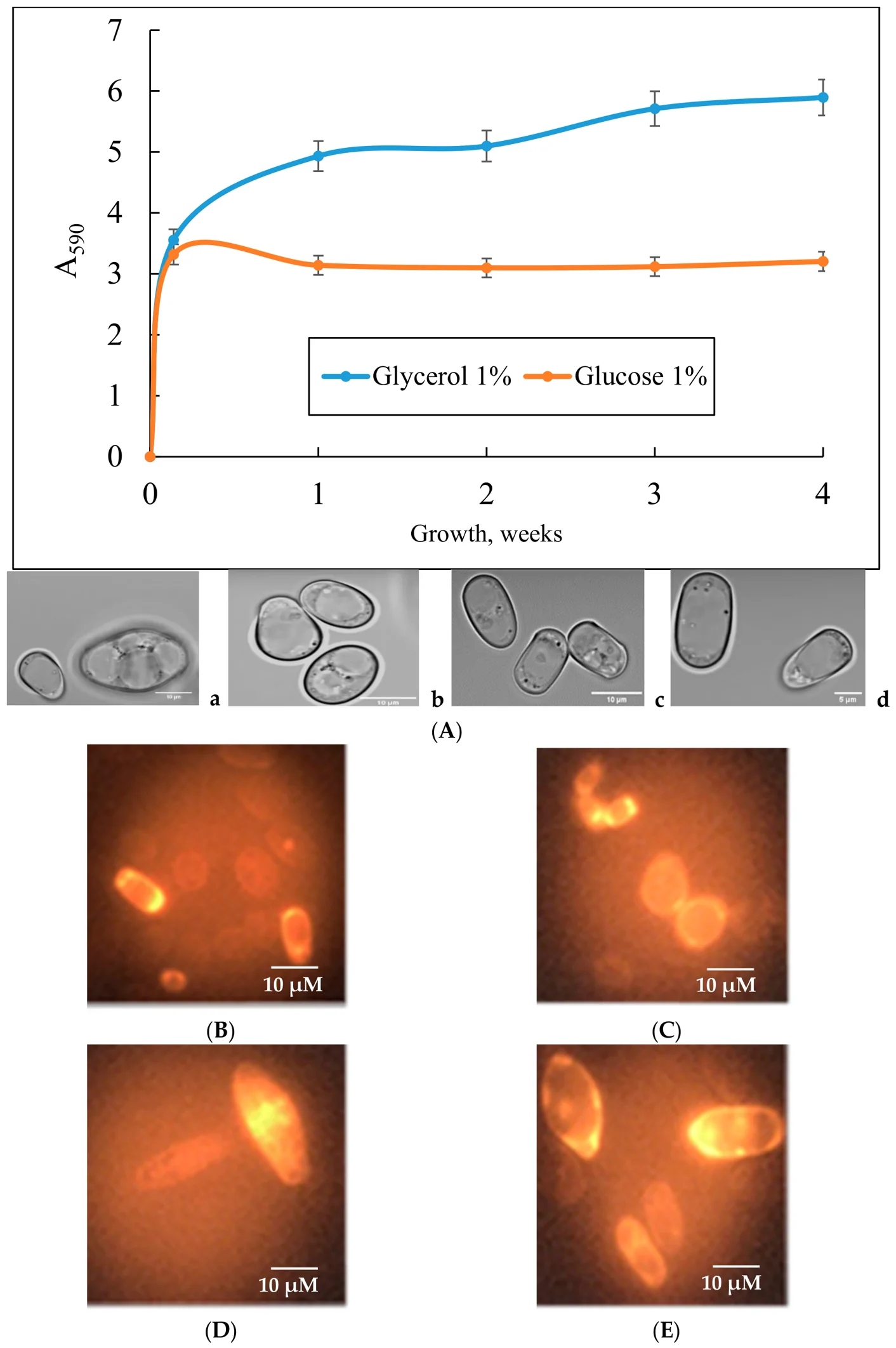
Micro-images of the *E. magnusii* cells (**B**–**E**) and growth curves of the cultures upon growth using different carbon sources (**A**). (**A**(**a**,**c**))—The cells at the one-week (**a**) and four-week (**c**) growth stages upon cultivation on glycerol; (**A**(**b**,**d**))—the cells at the one-week (**b**) and four-week (**d**) growth stages upon cultivation on glucose. Potential-dependent staining of mitochondria in the *E. magnusii* cells by MitoTracker Red (**B**–**E**). The cells were incubated with MitoTracker Red for 20 min. The incubation medium contained 0.01 M PBS, 1% glycerol, pH 7.4. The areas of high mitochondrial polarization are indicated with bright-red fluorescence due to the concentrated dye. The cells were visualized with a fluorescence microscope (at 100×), using a blue UV filter: (**B**,**C**)—glycerol, 1%; (**D**,**E**)—glucose, 1%; (**B**,**D**)—one- week growth stage; (**C**,**E**)—four-week growth stage.

E. magnusii can utilize various substrates. Thus, E. magnusii VKM U-261 can assimilate sucrose and raffinose but cannot grow using D-xylose. We studied the growth dynamics of E. magnusii on an “oxidative” type of substrate, glycerol, and a “fermentation” type of substrate, glucose. Figure 1A shows that the growth curves of the E. magnusii yeast varied significantly depending on the substrate type. Growth on 1% glycerol, an “oxidizing” substrate, led to a steady accumulation of biomass for about 20–24 h until the stationary growth stage and continued for another 16 h for a total of 36 h (Figure 1A), followed by permanent growth for four weeks (Figure 1A). The growth pattern on a “fermentative” substrate (1% glucose) was very different from its growth on glycerol. The stationary growth phase lasted for the same amount of time as the yeast growing on glycerol. However, at the same time, the maximum biomass yield in the stationary cultivation stage was almost two times lower than during glycerol utilization (Figure 1A). The stationary phase under these conditions was not accompanied by any changes in culture density, except for a slight drop in the stage from 24 to 168 h of growth (Figure 1A(a,b)). At the initial stages of cultivation, cellular organelles and small vacuoles were clearly visible. The degree of cell vacuolization increased with age (Figure 1A(c,d)).

Of note, the batch cultures in the experiments have not been replenished. Moreover, in the conditions of controlled evaporation throughout the whole experiment, the liquid loss reached not more than 10% of the initial volume (up to 90 mL). When grown using glycerol, at the 24 h growth stage, the volume of the cultural medium was 97.5 mL; at the one-week growth stage it decreased to 94.0 mL, in two weeks it decreased to 92.0 mL, in three weeks 90.0 mL, and in four weeks 89.8 mL. In the glucose-assimilating populations, at the 24 h stage the cultural medium had decreased to 98.5 mL; in one week it reached 98.0 mL, in two weeks it reached 94.0 mL, in 3 weeks it decreased to 92.0 mL, and in four weeks 90.0 mL. The pH of the medium changed according to the graph in Figure S1. The figure shows that for the first day of cultivation, the pH of the cultural medium dropped to 4.0, followed by some increase within two weeks. The value of pH nearly completely recovered to the initial level by the end of the experiment, being equal to 5.3–5.4. The acidification of the medium may be associated not only with the depletion of the carbon substrate, but also to the consumption of some medium components contributing significantly to pH homeostasis, for example, the phosphates.

We assayed the glycerol and glucose amounts in the cultural medium during the yeast cultivation using gas–liquid chromatography. Chromatographic data on the depletion of growth substrates in the culture medium throughout the whole experiment are presented in Table S1 and Figure S2. A complete depletion of glucose in the growth medium happened already on the first day of the experiment (Table S1, Figure S2), while upon glycerol assimilation at the 24 h growth stage, the remaining substrate in the medium accounted for about 32% of the initial level; however, at the one-week growth stage, its complete depletion was observed (Table S1, Figure S2). The changes in the substrate level provide quite convincing evidence that the yeast population, beginning from the second day for glucose and from the second week for glycerol, is in a state of carbon-depleted starvation, and indeed represents aging cultures.

Potentiometric staining of the *E. magnusii* cells with the MitoTracker™ Red CMXRos probe, which recorded the dynamics of the mitochondrial membrane potential, revealed a high-energy potential in all the cultures studied upon growth on different substrates and at different growth stages (Figure 1B–E). The cells grown using 1% glycerol were oval, ovoid, or cylindrical and up to 30 microns long and 10 microns wide (Figure 1B,D). These were nearly 1.5-fold smaller than the glucose-assimilating cells (Figure 1C,E). Most cells using 1% glucose were of slightly elongated and cylindrical shape, and were up to 40 × 15 microns in size (Figure 1C,E). As the population aged, the cells were a bit shortened (Figure 1). It is of note that even at the four-week growth stage, all the cultures studied showed dividing and budding cells (Figure 1).

**Figure 2:**
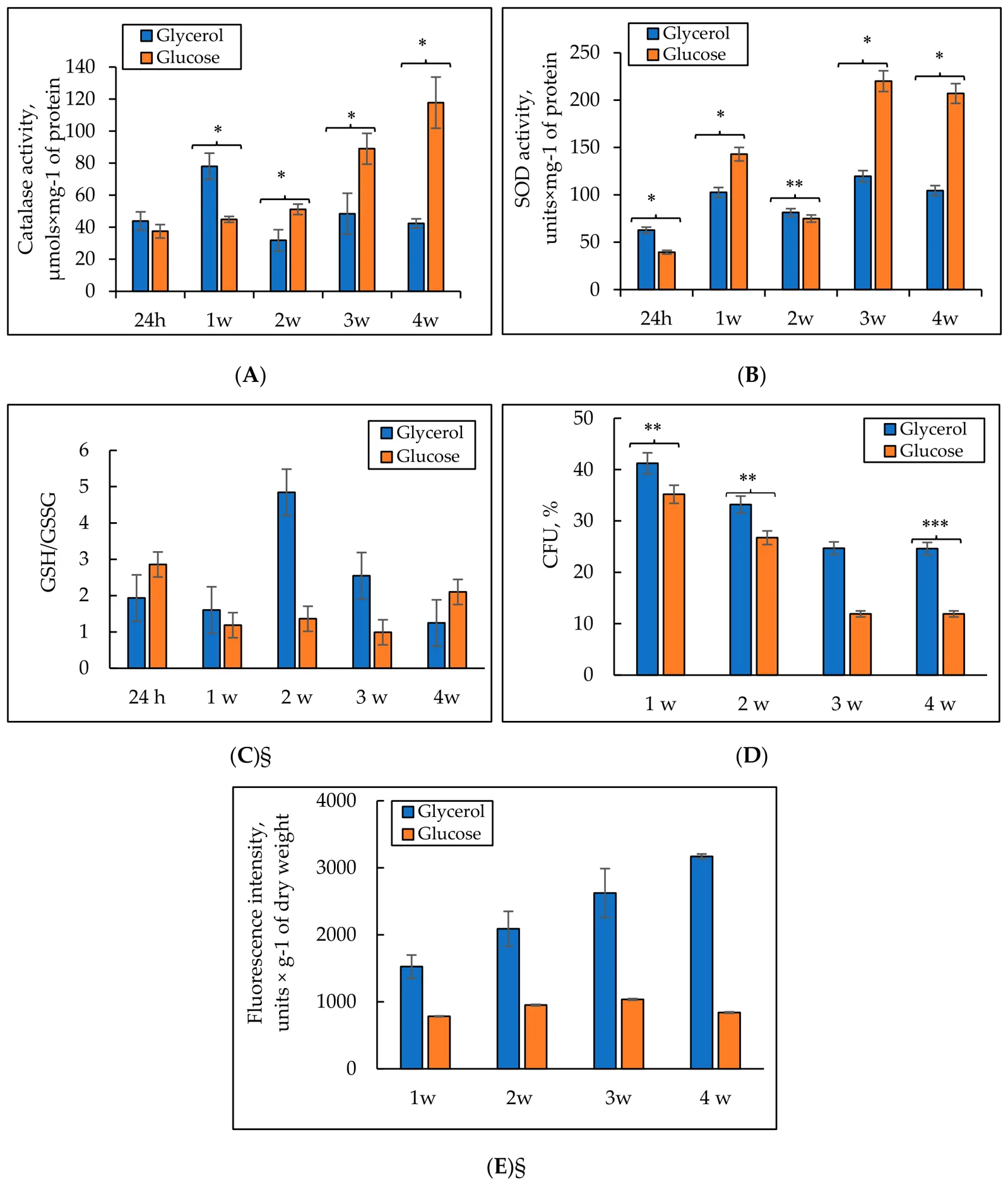
Antioxidant status and colony formation capacity in *E. magnusii* yeast utilizing various substrates. (**A**) Total CATs activity was assessed by monitoring the cellular homogenate at 240 nm with a Specol-11 spectrophotometer (Carl Zeiss Industrielle Messtechnik GmbH, Oberkochen, Germany). The procedure was performed in 20 mM KP_i_ buffer, pH 7.2, containing 2 mM EDTA. The reaction was triggered by the application of H_2_O_2_ (the molar extinction coefficient 46.3 M^−1^ cm^−1^). The CATs activity was measured by monitoring the decrease in hydrogen peroxide concentration. A_240_ was determined every 15 s for 3 min after the reaction started. CATs activity was expressed in μmols used H_2_O_2_ × min^−1^ × mg^−1^ of protein. (**B**) The activity of SODs in the cell suspension was assessed by monitoring of A_406_ with a spectrophotometer upon inhibition of quercetin autooxidation. The procedure was performed in 20 mM KP_i_ buffer, pH 10.2, containing 0.8 mM TMDA, 0.1 mM EDTA, and 1.4 µM quercetin. A 50% inhibition of quercetin autoxidation was considered as a SODs enzymatic activity unit. (**C**) To determine the ratio of reduced to oxidized glutathione, the reduced and oxidized glutathione was assayed according to the reaction with ortho-phthalic aldehyde (see the Section 2). (**D**) To determine the number of colony-forming units (CFU), we calculated the number of CFU as the number of reproductively capable cells in a sample as follows: number of CFU × dilution × factor × 10 = number of reproductively capable cells per mL (see the Section 2). (**E**) To determine the ROS level in the glycerol and glucose-assimilating *E. magnusii* yeast, raised at different growth stages, the dynamics of intracellular ROS production were monitored using a spectroscopic fluorescence probe of dihydro-2’,7’-dichlorofluorescein diacetate ester H_2_DCF-DA. Values are mean ± S.E.M from 5 to 6 independent experiments. Error bars represent the standard deviation of triplicates to each experiment. *—*p* < 0.01; **—*p* < 0.05; ***—*p* < 0.02. §—Statistically significant difference, *p* < 0.005. n = 5.

**Figure 3:**
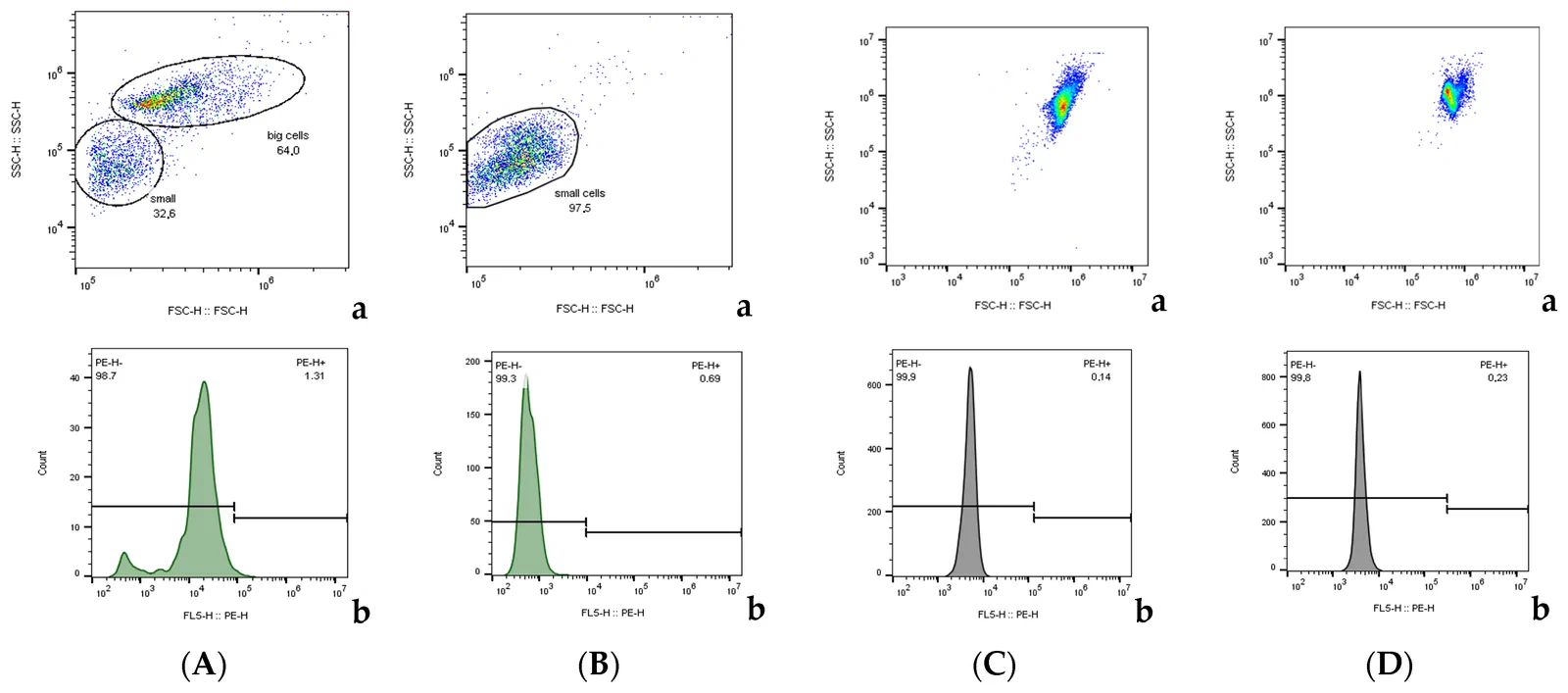
Distribution histograms of the *E. magnusii* yeast at the different growth stages using flow cytometry. The cells are stained with PI (see Materials and Methods for details). Notations: Upper panels (**a**) show the cell size distribution. X-axis (FSC-H)—forward scatter, y-axis (SSC-H)—side scatter. Bottom panels (**b**) show the cytogram of PI/FITC-PNA staining. The cells can be divided into live/non-live according to their green or red PI fluorescence. The x-axis (FL5-H) is the PI fluorescence intensity; the y-axis is the number of cells tested. (**A**,**C**)—The glycerol-assimilating cultures; (**B**,**D**)—the glucose-assimilating cultures; (**A**,**B**)—18 h of growth; (**C**,**D**)—four weeks of growth.

Nevertheless, while assaying the yeast population survival upon long-term cultivation, we showed that the ability to form colonies decreased significantly with age, regardless of the substrate type; however, glycerol-utilizing yeast were of higher potential for CFU formation, two-fold more than that in the cells growing on glucose (Figure 2D). Moreover, the majority of the *E. magnusii* cells in all the versions tested showed high survival according to staining with vital dyes and PI (Figure 3). In the glycerol-assimilating cultures, 98–99% of the cells showed negative staining throughout the whole experiment (Figure 3). The data indicate that the substrate significantly increased CLS.

Analysis of the redox status of yeast cells, including assessment of catalase (CATs), SODs, the ratio of reduced and oxidized glutathione, and ROS generation showed that the maximum activity of CATs and SODs in the cells grown on glycerol occurred at the one-week growth stage, while when cultured on glucose, the activity of both enzymes increased with age (Figure 2A,B). It should also be emphasized that the ROS level was more than two times higher during glycerol utilization in relation to glucose (Figure 2E), which corresponded to the role of this “oxidative” type of substrate in the induction of mitochondrial metabolism, activation of the full-fledged operation of the electron transport chain, and generation of oxygen radicals. At the same time, the ratio of reduced and oxidized forms of glutathione was significantly higher in cells growing on glycerol at the 2- and 3-week growth stages (Figure 2C). Thus, the redox status of E. magnusii cells indicated the development of moderate oxidative stress accompanied by an adaptive response of cellular systems.

### 3.2. Changes in the Metabolic Profile of the E. magnusii Yeast upon Prolonged Cultivation

A high survival rate and energetic activity of the *E. magnusii* population in the late stages of growth and in the deep stationary phase prompted us to study cell metabolic changes throughout the whole experiment. Metabolomic profiling resulted in annotating 162 peaks of the compounds found in three or more samples. The peaks found only in one or two samples were excluded from the following consideration. The metabolic matrix included the compounds of the following classes: carbohydrates, carbohydrate lactones, carbohydrate acids, deoxy sugars, phospho-sugars, polyols, polyols phosphates, amino acids, ribonucleosides, pyrimidine nucleosides, fatty acids, inorganic acid, fatty acid derivatives, glycerolipids, monoacylglycerols, sterols, carboxylic acids, dicarboxylic acids, tricarboxylic acids, hydroxy carboxylic acid, carboxylic esters, hydroxy carboxylic acids, glycosides, *o*-glycosides, and phenols (Table S2). About 20% of the substances’ classes could not be identified.

**Figure 4:**
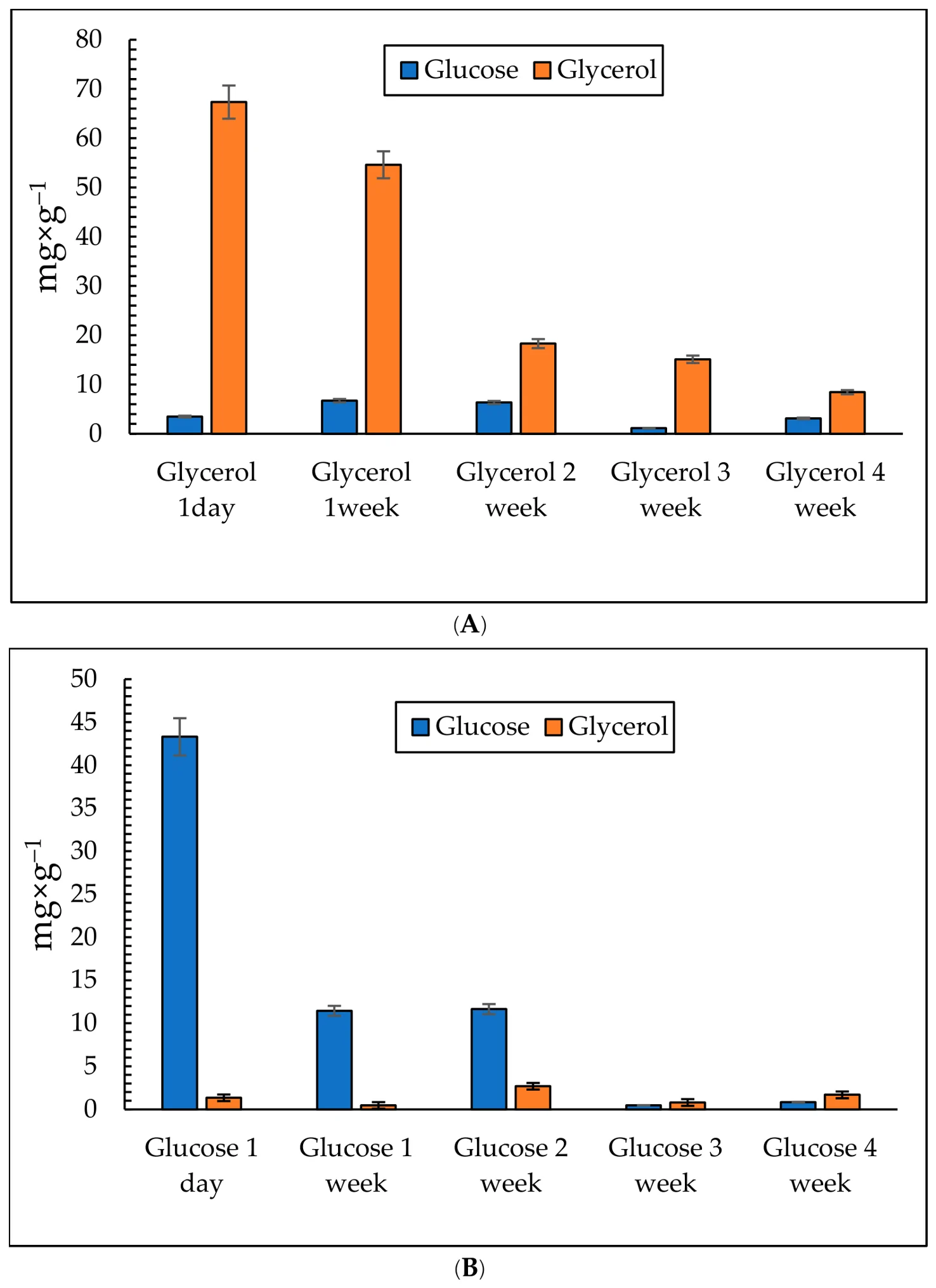
Dynamics of glucose and glycerol content in *E. magnusii* cells grown on glycerol (**A**) and glucose (**B**) as growth substrates during long-term cultivation. The results were obtained using GC-MS; quantitative analysis was performed taking into account sensitivity coefficients obtained for standard compounds (glycerol and glucose) using the same method. The experiment was performed in triplicate. The results represent the mean value ± the standard error of the mean.

Quantitatively, polyols and sugars dominated in all the samples tested. Among the polyols, arabitol, glycerol, mannitol, and myo-inositol showed the highest concentrations. The concentrations of erythritol and sorbitol were two- to three-fold lower. Galactinol, xylitol, *D*-ribo-hexitol, and 3-deoxy showed even lower levels (by 10–30 times). Glucose, 2-deoxyglucose, ribose, lyxose, galactose, and galactono-1,4-lactone prevailed among monosaccharides. Among the disaccharides, trehalose and maltose dominated. Moreover, a tetrasaccharide of stachyose showed fairly high levels. The concentrations of mono-, di-, and tetrasaccharides reached 3.4% of the total biomass (Table S1). The concentrations of the other compounds were significantly lower. The initial substrates, namely glucose and glycerol, were quantified using calibration according to the standards and calculation per biomass (Figure 4). As for the other compounds, a semi-quantitative assay was performed to track the concentration difference in the metabolites.

The metabolite levels significantly depended on the carbon growth source and the growth stage. To visualize the metabolomic differences in the culture at the different growth stages, some statistical PC models were designed for cultivation on glycerol and glucose.

#### 3.2.1. Glycerol

**Figure 5:**
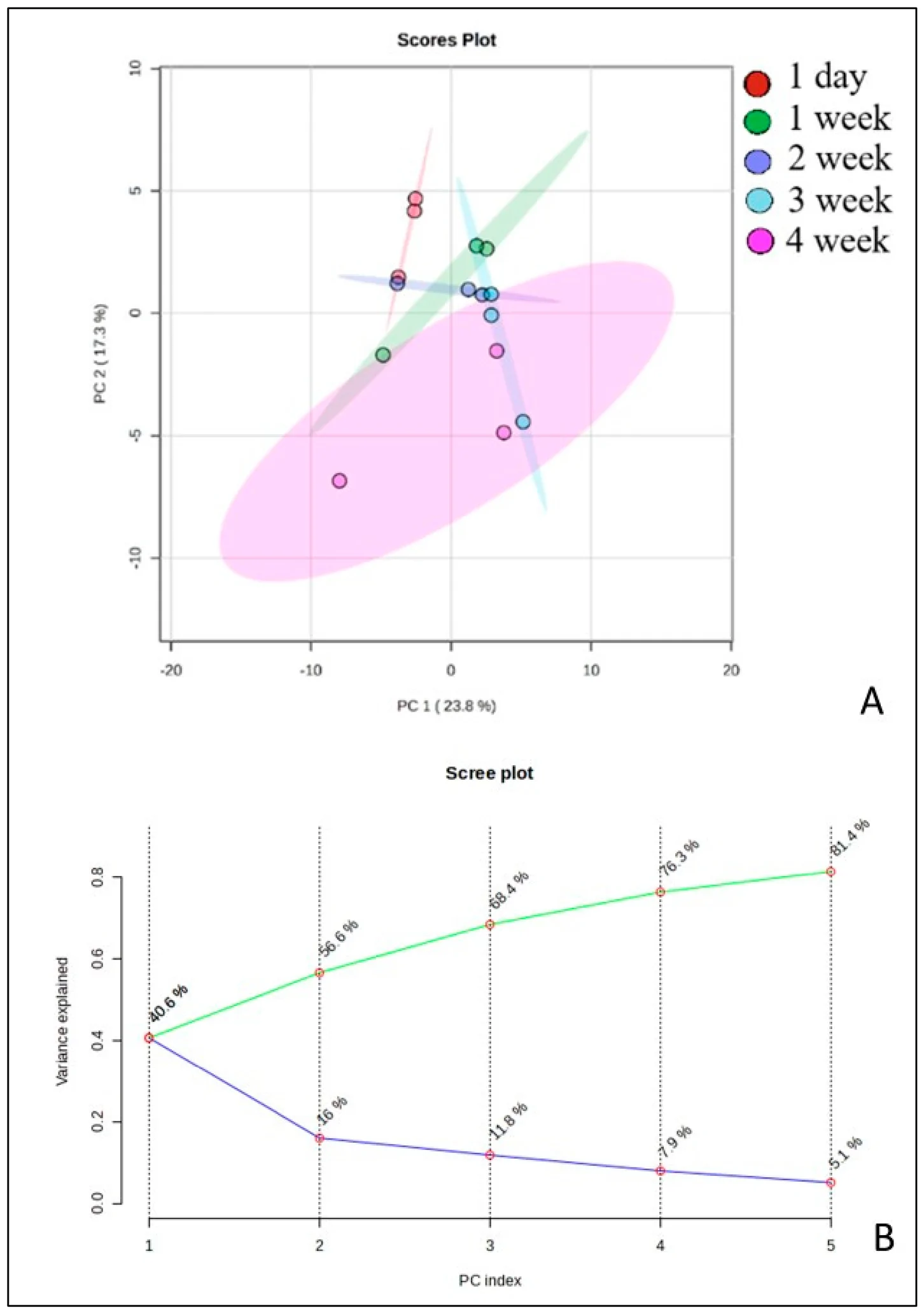
PCA of the metabolome of yeasts that metabolized glycerol. (**A**)—PCA score plot between component 1 versus component 2 (F-value: 4.8394; R-squared: 0.65937; *p*-value (based on 999 permutations): 0.004). (**B**)—Scree plot was generated for the first five principal components (PCs) to identify the contribution of each of the principal components to the total variation in the data set. The green line shows the cumulative variance explained by the first five components, which accounted for 56%, whereas the blue line indicates the individual variance for each principal component.

According to the data in Figure 4A, the glycerol amount gradually declined upon culture growth, reaching its minimum by the fourth week of cultivation. However, the glucose level of the yeast grown on glycerol showed more complicated dynamics: it doubled at the two-week growth stage, then decreased dramatically by the third-week stage, before restoring its initial level at the four-week stage of growth (Figure 4A). Figure 5 shows the PC model for the cultures grown on glycerol, where in its A part (score plot) the samples were clustered according to the growth stage. The model parameters (F-value: 4.8394; R-squared: 0.65937; *p*-value (based on 999 permutations): 0.004) indicated its significance (*p* < 0.005). Main components 1 and 2 described 56% of the total variance (Figure 5B). The groups of samples at the early growth stages showed the lowest dispersion in the main components space, while the late stages of growth (three and four weeks of cultivation) showed the highest dispersion.

**Figure 6:**
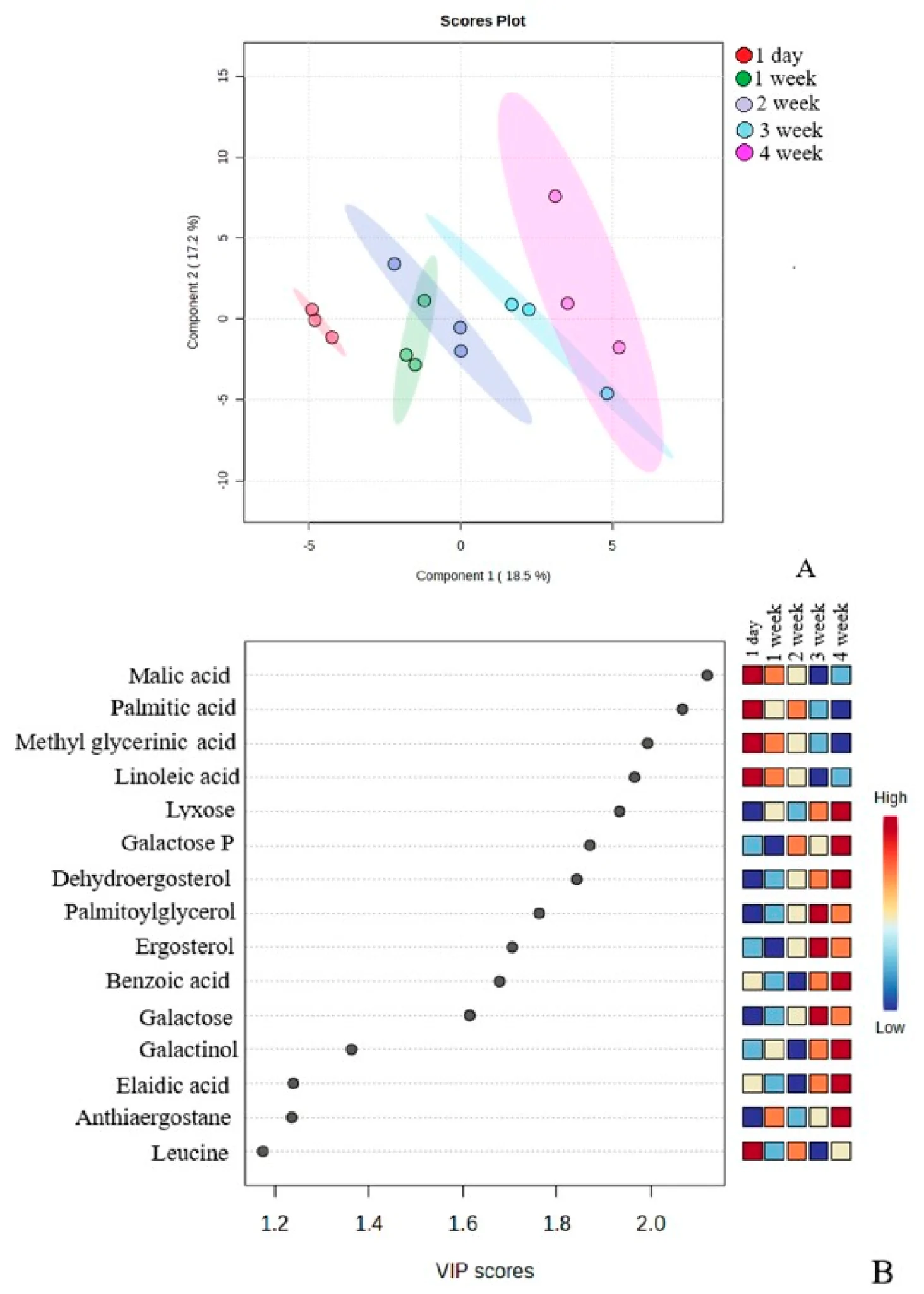
PLS-DA of the metabolome in the population metabolizing glycerol. (**A**) PLS-DA score plot of all the metabolites tested; (**B**) feature importance. The PLS-DA model showed moderate explanatory power (R^2^ = 0.74) and acceptable predictive power in cross-validation (Q^2^ = 0.53). Despite moderate overfitting (0.21 gap), the model is statistically significant (permutation test, *p* < 0.05) and can be used to select potential metabolic markers.

Partial least squares discriminant analysis (PLS-DA) was used to identify the typical metabolites, which accumulate in the yeast population at the different growth stages. The PLS-DA model allows us to determine the variable metabolites contributing the greatest difference in the samples by group (Figure 6, Table 1). Factor loads of the predictive component and Variable Importance Plot (VIP)-estimates of metabolites (values more than 1) (the importance of the variable in the projection) were used to assess the statistical relationship between variables and the factor of interest (the growth stage). Figure 6B shows that the organic acids (malic, methylglycerol, palmitic, linoleic, and elaidic) made the greatest contribution to data clustering. The VIP metabolites group also included some representative of the glyceride class, namely palmitoylglycerol, sterols (ergosterol and dehydroergosterol), monosaccharides (lyxose and galactose), polyol galactinol, phenolic compound anthiaergestan, aromatic carboxylic acid benzoic acid, and amino acid leucine.

**Figure 7:**
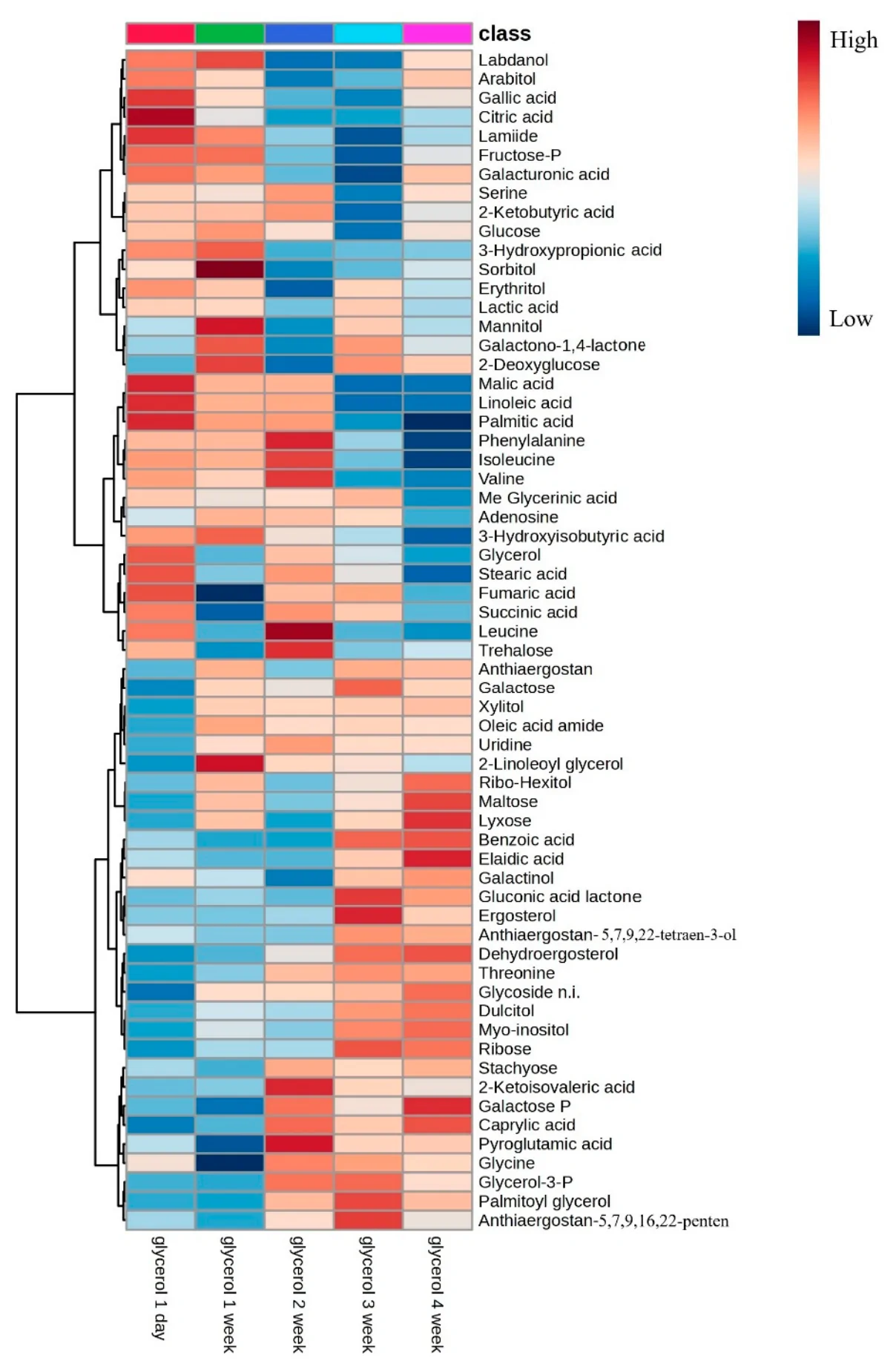
Heat map of the metabolome profile for the yeast grown on glycerol.

Figure 7 shows a heat map combined with a hierarchical clustering dendrogram (distance is 1-r, Ward’s method) of all the metabolites identified in the yeast cultivated on glycerol. The metabolites were clustered into two large groups: the first one, which mainly accumulated at the beginning of cultivation (from the first day until two weeks), and the second, which did so in the second half (from two to four weeks of growth). The first group included amino acids (leucine, isoleucine, valine, phenylalanine, serine), carboxylic acids of energy metabolism (citric, malic), fatty acids (linoleic acid, palmitic acid, 2-ketobutyric acid), carbohydrates, their derivatives (glucose, fructose-P), and polyols (mannitol, arabitol, and glycerol). The concentrations of some compounds showed more complicated dynamics. Thus, the amount of fumaric, succinic, and stearic acids decreased in the first week of growth, then increased again in the two- and three-week-old population and declined significantly by the fourth week of cultivation (Figure 7). Some compounds level (benzoic and elaidic acids) decreased upon the cultivation, but increased slightly in the aging culture (Figure 7). The second cluster included the compounds increasing their level during the ontogenesis. The group included sterols (egrosterol and dehydroergosterol), most polyols (xylitol, galactinol, dulcitol, myo-inositol), sugars and their derivatives (maltose, stachyose, lyxose, ribose, galactose-P), uridine, amino acids (threonine, pyroglutamic acid, glycine), benzoic acid, anthioergostanes, lactone of gluconic acid, anthioergostan, glycoside, caprylic acid, oleic acid, amide, and monoacylglycerols (Figure 7).

#### 3.2.2. Glucose

Figure 4B shows the dynamics of the glucose and glycerol amounts in *E. magnusii*, a yeast assimilating glucose. The glucose level sharply declined, reaching its maximum in the culture at the three-week-old stage and then nearly doubled by the fourth week **(**Figure 4B). However, the glycerol level upon growth on glucose showed complicated dynamics; namely, at the two-week-old growth stage it doubled compared to that in the one-day-old culture, then crucially declined at the three-week-old growth stage and restored the initial level by the end of cultivation (Figure 4B).

**Figure 8:**
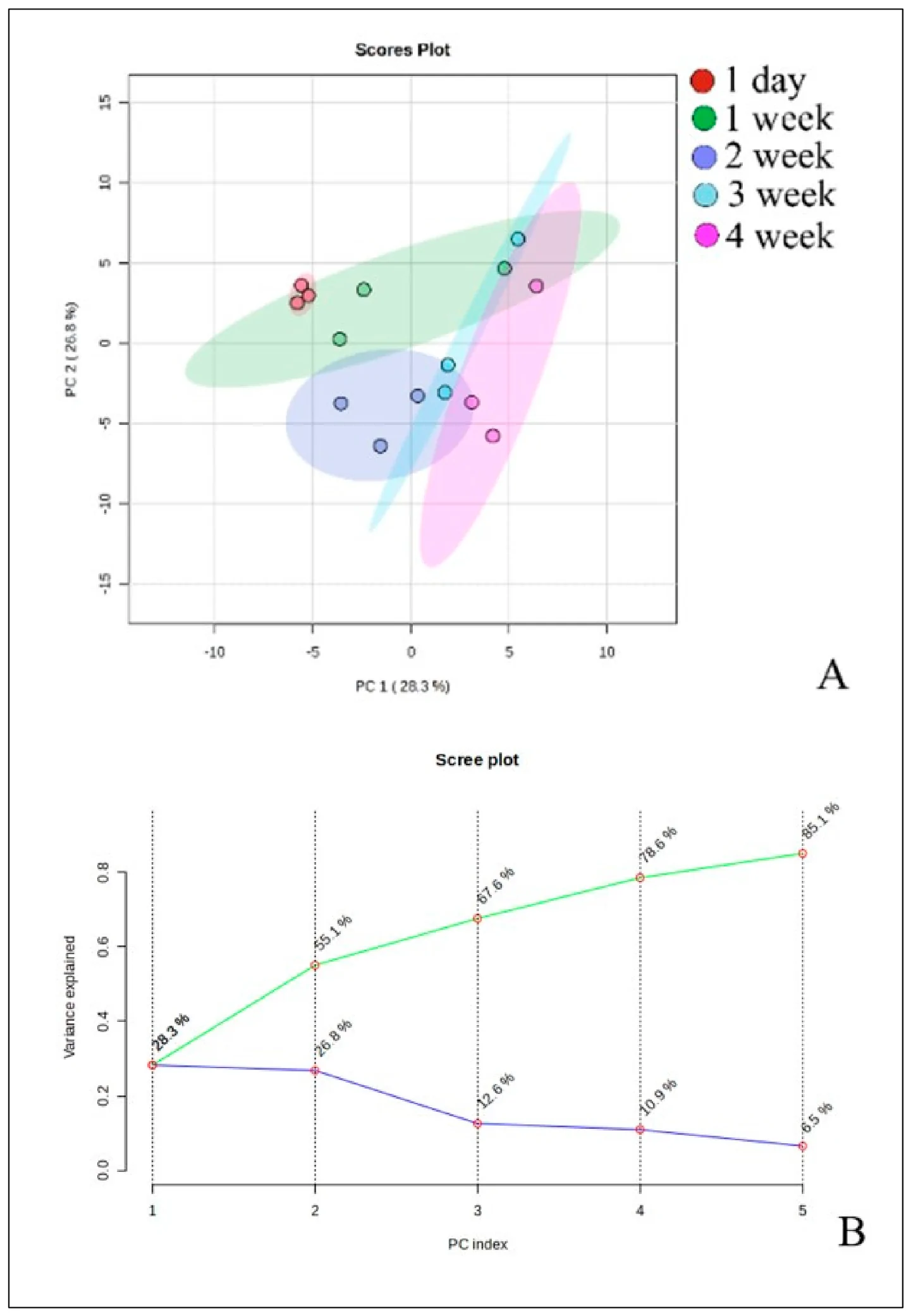
PCA of the metabolome of yeasts metabolized glucose. (**A**)—PCA score plot between component 1 versus component 2 (F-value: 4.8582; R-squared: 0.66024; *p*-value (based on 999 permutations): 0.003). (**B**)—Scree plot was generated for the first five principal components (PCs) to identify the contribution of each of the principal components to the total variation in the data set. The green line shows the cumulative variance explained by the first five components, which accounted for 55%, whereas the blue line indicates the individual variance for each principal component.

The dynamics of accumulation of the metabolites typical to the growth of *E. magnusii* upon assimilating glucose significantly differed from those on glycerol. The PCA model (Figure 8) indicated that the samples were clustered depending on the growth stage, although this clustering was less pronounced than that while growing on glycerol. The model parameters (F-value is 4.3469; R-squared is 0.63487; *p*-value (based on 999 permutations) is 0.005) testify to its significance (*p* < 0.005). Main components 1 and 2 described 56% of the total variance (Figure 8B). The group of one-day-old samples showed the lowest variance in the space of the main components.

**Figure 9:**
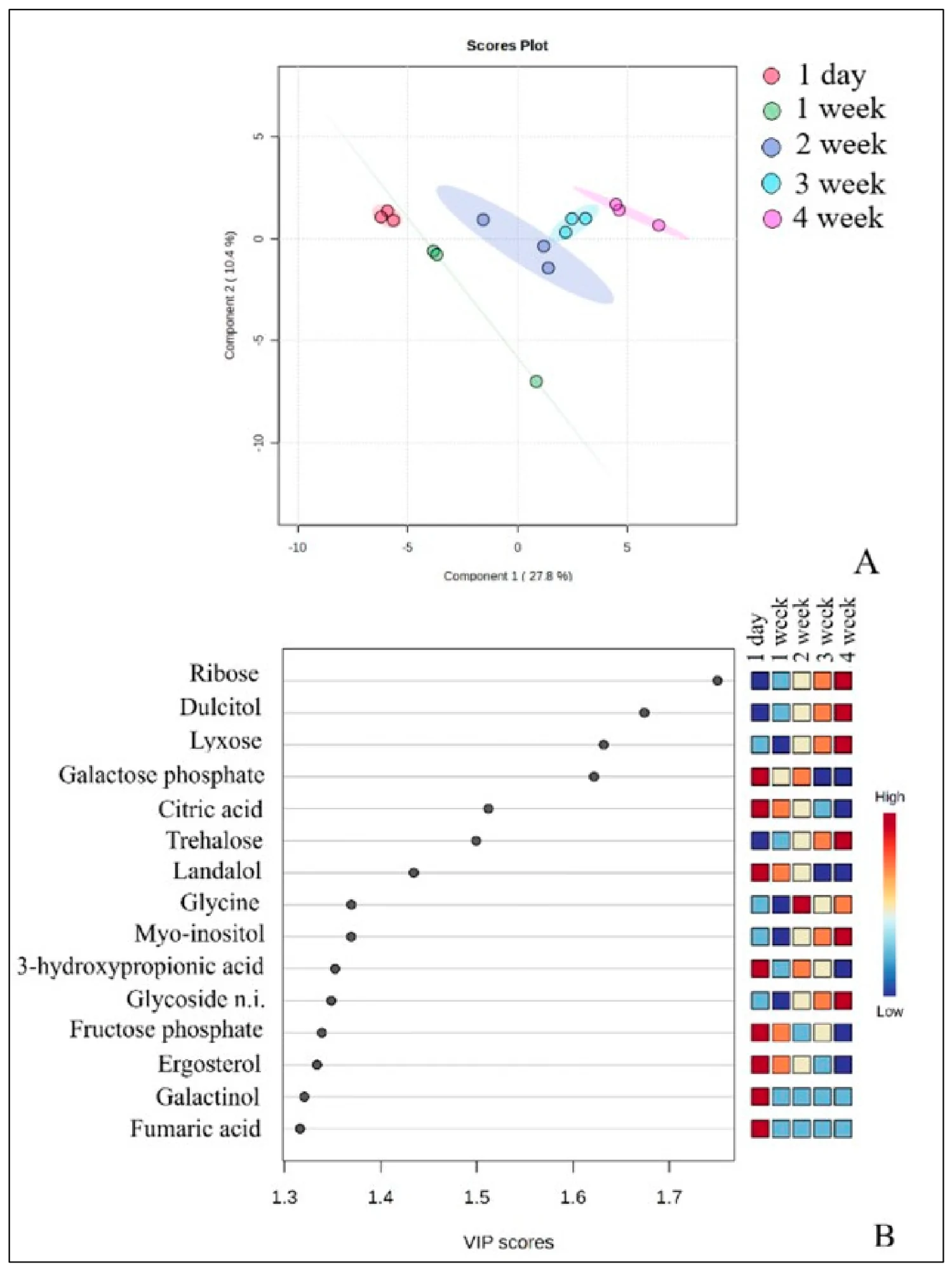
PLS-DA of the metabolome of the yeasts metabolizing glucose. (**A**) PLSDA score plot of all the metabolites tested; (**B**) feature importance. The PLS-DA model demonstrated high explanatory power (R^2^ = 0.91) and excellent predictive power in cross-validation (Q^2^ = 0.79). The small difference between R^2^ and Q^2^ (gap of 0.12) indicates minimal overfitting and good generalization ability of the model. The permutation test confirmed statistical significance (*p* < 0.05). The model is robust and interpretable.

According to the PLS-DA model (Figure 9, Table 1), the group of VIP metabolites included mainly sugars, polyols, and organic acids, being the products of direct sugar oxidation (Figure 9B). Moreover, it included hydroxypropionic and citric acids, and the amino acid glycine.

**Figure 10:**
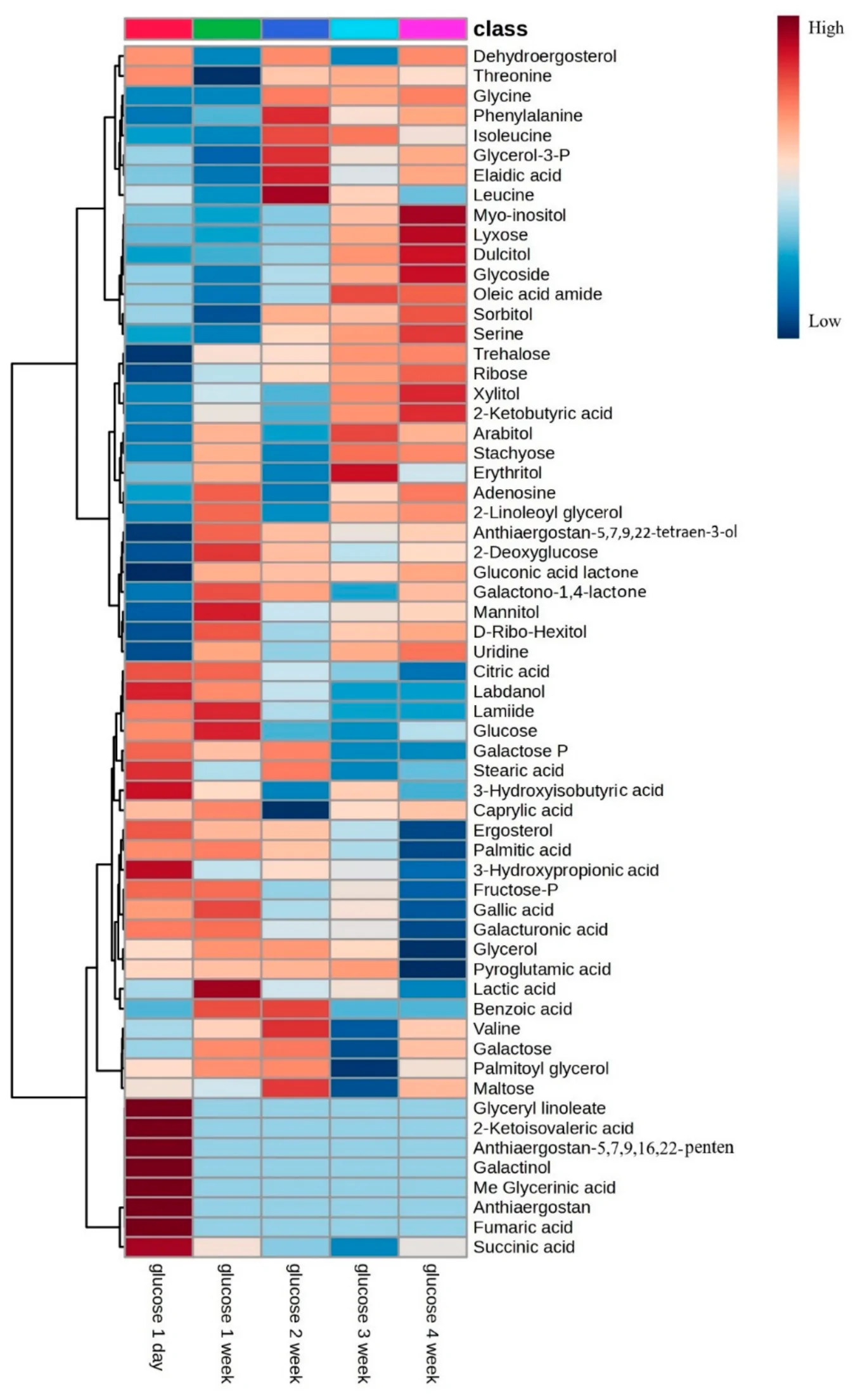
Heat map of the metabolome of the yeast assimilating glucose.

The heat map (Figure 10) shows that, like in the case of glycerol, the metabolites were clustered into two large groups: the first one displayed the main accumulation at the beginning of cultivation (from 1 day to 2 weeks), and the other in the second half of ontogenesis (from 2 to 4 weeks). A subgroup of identified compounds in the first cluster had the maximum concentration on the first day of growth, which decreased significantly in the week-old culture. The subgroup consisted of organic acids (fumaric acid, succinic acid, hydroxyisobutyric acid, hydroxypropionic acid), galactanol, glyceryl linoleate, anthioergostan 5,7,9,16,22—penten, Me glycerol acid, and 2–ketoisovaleric acid (Figure 10). The amount of lamiide and labdanol reached their maxima in the first week of growth (Figure 10). The level of galactose-P, fructose-P, citric acid, ergosterol, galacturonic acid, palmitic acid, gallic acid, and pyroglutamic acid decreased more gradually. This cluster also included some compounds with more complicated dynamics. Thus, the level of stearic acid reached its maximum on the first day of growth, then decreased slightly and increased again by the second week of cultivation (Figure 10).

The second cluster included the compounds with increasing concentration upon cultivation. The cluster also included two main subgroups. The highest level of the substances of the first subgroup accumulated in the second and third weeks of growth. The group included most of the amino acids (phenylalanine, valine, leucine, isoleucine, and pyroglutamic acid), polyols (erythritol, arabitol), sugars (maltose, galactose), glycerol-3-P, elaidic acid, galactonic acid lactone, and benzoic acid (Figure 10). The second subgroup included the compounds accumulating at maximal concentrations in the fourth week of cultivation. It included polyols (myo-inositol, dulcitol, xylitol, sorbitol), sugars (trehalose, ribose, stachyose), amino acids glycine and serine, as well as ketoisobutyrate and uridine (Figure 10).

### 3.3. Assay of Differences in the Metabolomic Profiles of the E. magnusii Yeast Utilizing Various Substrates

**Figure 11:**
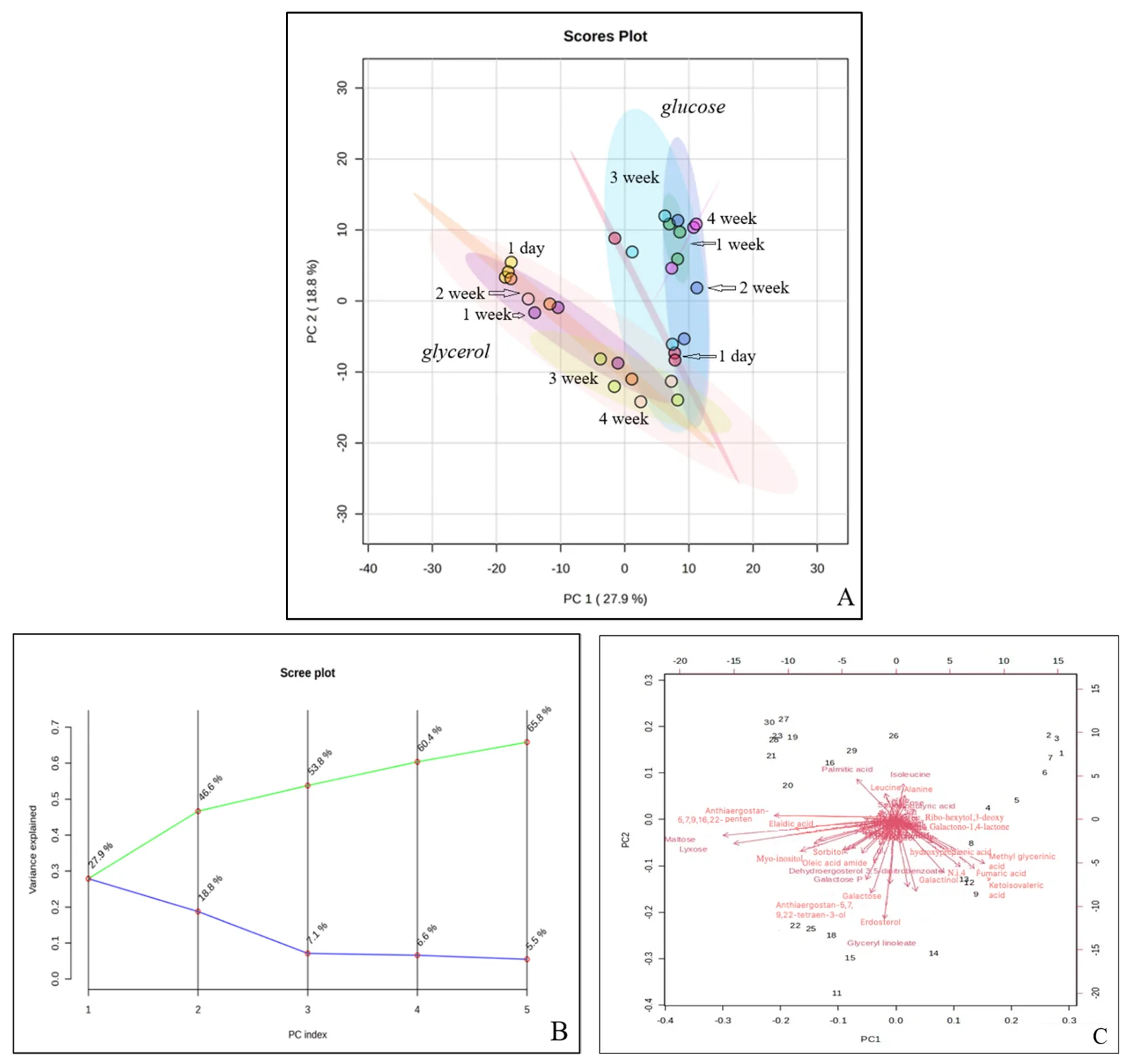
PCA of the yeast metabolome. (**A**)—PCA score plot between component 1 versus component 2 (F-value: 2.784; R-squared: 0.55611; *p*-value (based on 999 permutations): 0.002). (**B**)—Scree plot was generated for the first five principal components (PCs) to identify the contribution of each of the principal components to the total variation in the data set. The green line shows the cumulative variance explained by the first five components, which accounted for 66%, whereas the blue line indicates the individual variance for each principal component; (**C**)—PCA biplot between the selected PCs. Biplot identifying the relationship between samples and metabolites, where samples are displayed as points and metabolites are represented as vectors.

**Figure 12:**
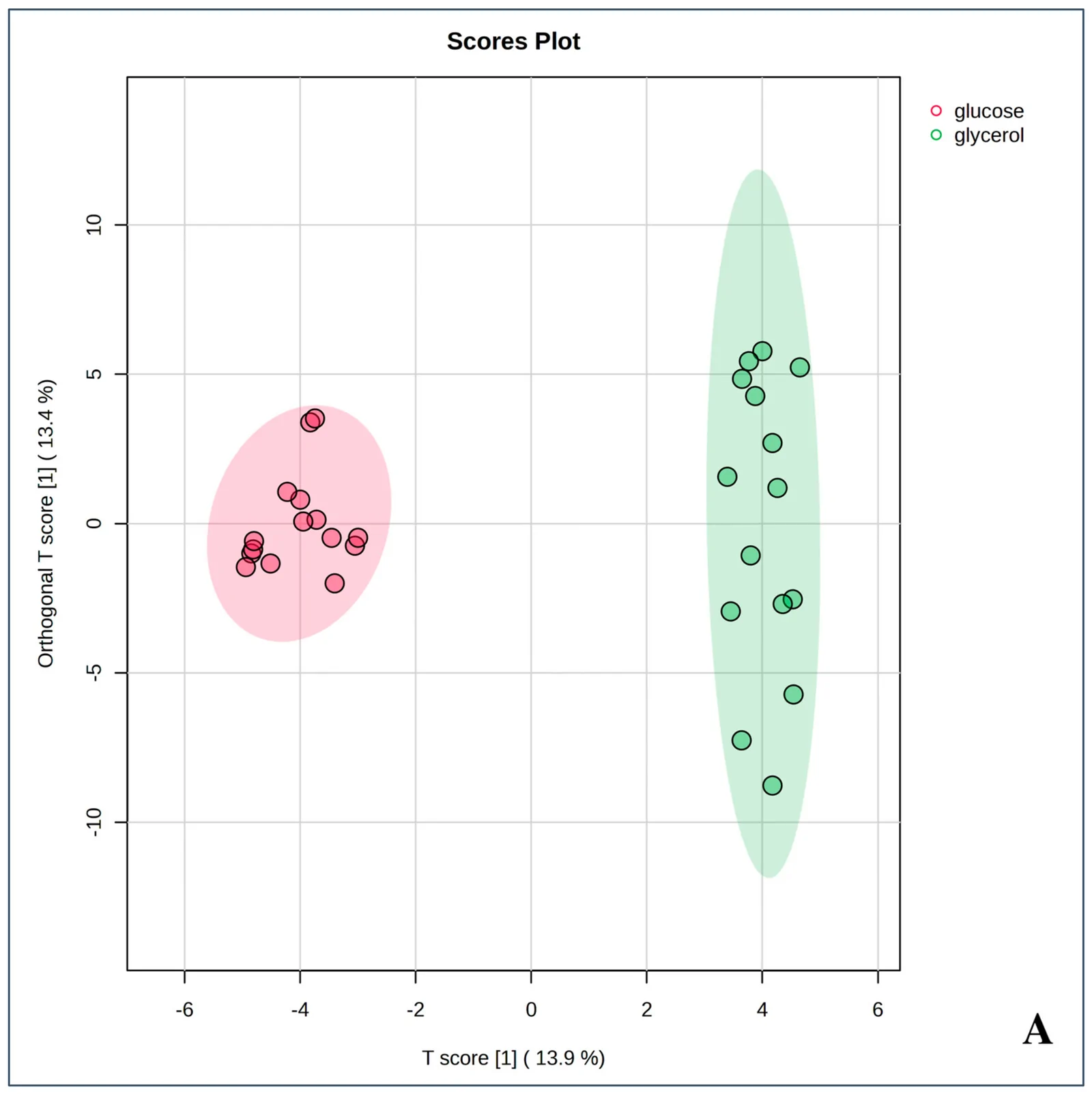

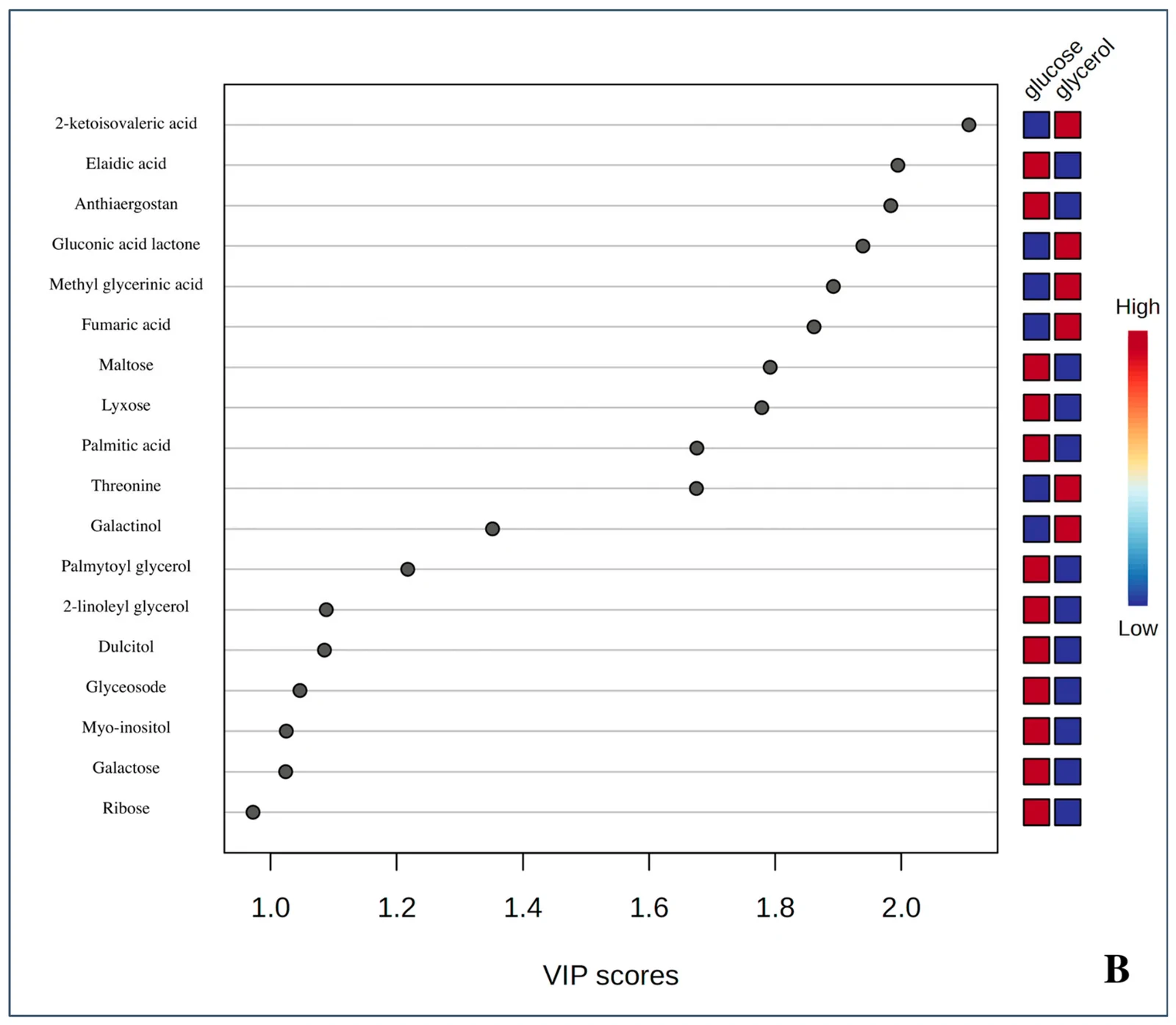
(**A**) OPLS-DA score plot of all the metabolites identified; (**B**) VIP scores of the significantly varying metabolites. The OPLS-DA model demonstrated good explanatory power (R^2^Y = 0.741) and high predictive power in cross-validation (Q^2^ = 0.669). The minimal discrepancy between R^2^Y and Q^2^ (gap of 0.072) indicates the absence of overfitting and good generalization ability of the model. A permutation test confirmed the statistical significance of the model (*p* < 0.05). The model is robust and suitable for selecting potential metabolic markers.

The analysis of the obtained data showed that the ontogenetic metabolic features of the yeast differ for the cultures assimilating either glycerol or glucose. The composition of the VIP metabolites depended on the carbon source. Galactose-P was the only compound in the VIP metabolites found in the cultures growing both on glucose and glycerol (Figure 6 and Figure 9). To assay the effect of the carbon source on the metabolic difference, PCA and PLS-DA analyses were performed (Figure 11 and Figure 12). The unsupervised PCA algorithm provides the possibility to achieve an objective dimensionality reduction, and its application reveals the group structure only if the variations within the group are significantly smaller than the variations between the groups. Initially, a PCA model was designed using five main components for 10 groups, i.e., each pattern (glycerol 1 day, glycerol 1 week, glycerol 2 weeks, glycerol 3 weeks, glycerol 4 weeks, glucose 1 day, glucose 1 week, glucose 2 weeks, glucose 3 weeks, glucose 4 weeks) of three samples (replicates) formed a separate group. No significant difference between the groups grown using the same carbon source in the model (*p* < 0.05) was revealed. Figure 11A evidently shows the difference between the samples with different carbon sources. According to the F.Model results, the largest data variance was obtained for the groups of one-week glucose and one-week glycerol stages. Components 1 and 2 contributed the most to the design of the model (Figure 11C). Load vectors of variables (metabolites) are shown in Figure 11C.

**Figure 13:**
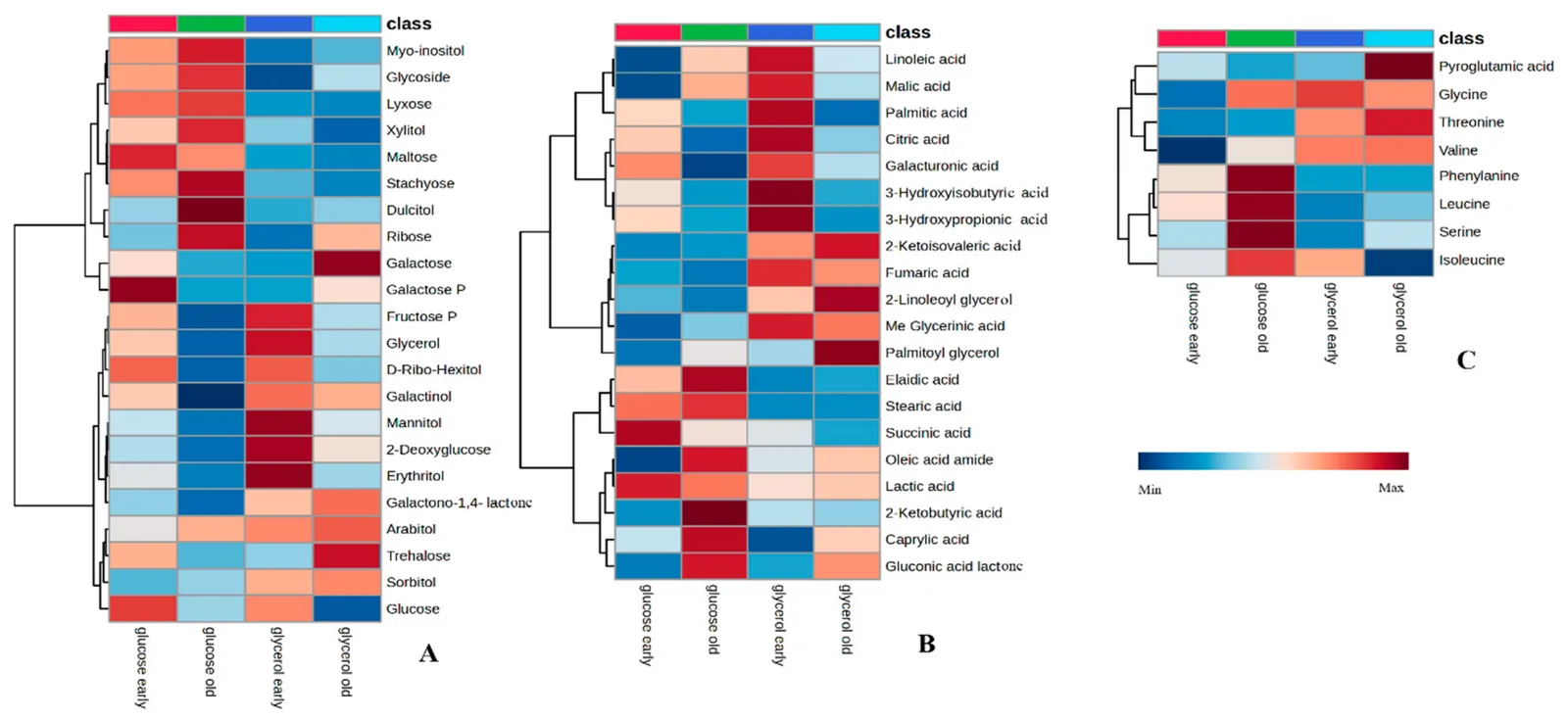
Heat map of the metabolites of various classes in the metabolic profile of yeast assimilating glycerol and glucose: (**A**)—sugars and polyols; (**B**)—organic acids and monoacylglycerols; (**C**)—amino acids. Early—1 day–1 week; old—2–4 weeks.

To compare the effect of glycerol or glucose on various metabolic links, heat maps were compiled combining the metabolic data obtained in the cases of glycerol and glucose for the compounds of three major classes, namely sugars and polyols, organic acids and monoacylglycerides, and amino acids. Unlike the previous visualizations (Figure 7 and Figure 10), the heat maps reflect no dynamics of metabolite accumulation. Nevertheless, for a more evident comparison, the data were divided into two groups depending on the stage of ontogenesis, namely the exponential (1 day–1 week) and the stationary (2–4 weeks) growth stages. Figure 13 shows how the carbohydrate level changes upon culture growth, and also depends on the initial carbon source (glycerol or glucose). Among the polyols, arabitol, mannitol, erythritol, and sorbitol dominated upon cultivation on glycerol, while dulcitol, xylitol, and myo-inositol clearly prevailed in the case with glucose. The carbohydrates of galactose, trehalose, and fructose phosphate dominated upon assimilating glycerol, while ribose, lyxose, maltose, galactose-P, and stachyose dominated if glucose was used as a carbon source. The metabolic profile of the yeast assimilating glucose and glycerol also included them, and their amounts were higher in the culture assimilating the appropriate substrates, especially on the first day of cultivation. Then, the amount of both substrates decreased upon growth.

The products of direct glucose and glycerol oxidation, methylglycerol acid and gluconic acid (as lactone), accumulated mainly in the cultures grown on glycerol and glucose, respectively (Figure 13B). The product of direct oxidation of galactose, namely galacturonic acid, was found in the metabolic profile of the cultures grown on both glycerol and glucose. Intermediate products of sugar oxidation, namely malic and citric acids, were revealed in the metabolic profile of the cultures grown on both types of carbon substrate, while fumaric acid was detected upon glycerol assimilation, and succinic acid prevailed in the yeast grown on glucose (Figure 13B). The product of the anaerobic sugar oxidation, lactic acid, prevailed in the glucose assimilating cultures (Figure 13B).

Monoacylglycerides (Figure 13B) predictably accumulated in the yeast upon assimilating glycerol starting from the first week of growth. In both cases, fatty acids, namely elaidic, caprylic, and stearic, accumulated mainly upon assimilating glucose, while palmitic, linoleic, and keto-isovaleric acids did so in the case of glycerol. Among the amino acids (Figure 13C), a certain specificity of the accumulation was also observed depending on the carbon substrate. Growth in the glycerol-containing medium led to a greater accumulation of pyroglutamic acid, threonine, and valine. However, cultivation on glucose resulted in the dominance of the amino acids phenylalanine, leucine, isoleucine, and serine, while glycine was found in both cases. Glucose is a more favorable carbon source for accumulating nucleosides, anthraergostans, and dihydroergosterol. Gallic acid and labdanol accumulated more when cultivated on glycerol.

## 4. Discussion

Metabolomics approaches are widely used on yeast objects as model organisms. So, some researchers studied the formic acid treatment on cellular metabolites of the *S. cerevisiae* yeast [57]. Sanchez et al. [58] described the metabolic reactions of the *Schizosaccharomyces pombe* fission yeast upon 24 h phosphate starvation. However, the application of metabolic research methods in the assay of yeast aging is extremely limited.

The studies by Titorenko’s group have shown that various inter-compartment communications in the yeast cell, namely organelle–organelle and organelle–cytosol interactions, play an essential role in yeast chronological aging [31,32,33]. The model was based on the assumption that the inter-compartment communications providing longevity include unidirectional and bidirectional movements of some metabolites between the mitochondria, cytosol, peroxisomes, nucleus, vacuoles, endoplasmic reticulum, plasma membrane, and lipid droplets. The authors suggested that the intracellular metabolite levels and/or the rate of their movement between cellular compartments undergo age-related changes. In the model, various changes in key metabolites were distinguished across several time periods that determine life span, and their spatiotemporal dynamics within cellular processes ensured the development and maintenance of a pro- or anti-aging cellular pattern [23]. The authors concluded that the main regulatory proteins specific to control points contribute to the design of a pro- or anti-aging cellular pattern, since each of the proteins modulates certain cellular processes determining life span.

In our study, first, we attempted to assay the metabolic profiles of the *E. magnusii* yeast using an “oxidative” substrate, glycerol, and a “fermentative” substrate, glucose, to search for possible key metabolites determining the chronological aging of the yeast population upon long-term cultivation.

### 4.1. Glucose Versus Glycerol: The Type of Carbohydrate Metabolism Matters

The influence of the nutrition type on yeast cultivation is crucial in predicting the rate and features of chronological aging. Glucose is a preferable substrate for most yeasts with the metabolism of “fermentative” type, in particular, the popular yeast *S. cerevisiae.* Thus, the utilization of this carbohydrate suppresses the expression of the genes responsible for the metabolism of the other carbon sources [59,60,61]. Moreover, yeast is capable of efficient aerobic growth on glycerol as a carbon source; transitions from “fermentative” to “respiratory” growth require significant transcriptional restructuring [62,63], which launches the genes encoding the elements of the respiratory chain, oxidative, osmotic, and general anti-stress responses, protein synthesis enzymes, glycerol utilization, and so on [64].

The results demonstrated that in cultures with glucose-based growth, their level gradually decreased with age (Figure 10), which agrees well with the logic of the fermentative type of substrate utilization and our previous data [37]. However, it should be noted that upon yeast growth, glucose concentration decreased sharply, reaching its minimum at the three-week growth stage, and then nearly doubled by the fourth week of cultivation (Figure 4B), which can be explained by some restructuring of metabolic pathways, probably associated with some activation of gluconeogenesis processes allowing the cells to maintain a metabolic state in the deep stationary stage. The induction of the gluconeogenesis and anaplerotic metabolic pathways is also confirmed by the appearance of methylglycerol acid, a metabolite associated with glycerol degradation, malic, and benzoic acids (Figure 6, Table 1) that indicates the involvement of polyol in gluconeogenesis via glyceraldehyde-3-phosphate; in other words, malic acid participates in the anaplerotic reactions to replenish oxaloacetate, while benzoic acid is a degradation product of aromatic amino acids, possibly happening upon starvation of the yeast cells in the deep stationary stage. A significant decrease in the fructose phosphate amount at the four-week stage compared to that at the three-week growth stage (Figure 10) also may indirectly confirm the assumption. Glucose levels in glycerol-assimilating cells decreased at the three-week growth stage and fully recovered to the initial level of carbohydrates by the fourth week of cultivation, indicating the switching of metabolic pathways to gluconeogenesis (Figure 4A). Thus, it can be concluded that at this growth stage, due to the high demand for energy sources, the gluconeogenesis pathway was induced via glycerol kinase and glycerol-3-phosphate dehydrogenase, which led to carbohydrate accumulation. In this reaction (conversion of glycerol-3-phosphate to dihydroxy-acetone), NADH is reduced, then oxidized in the mitochondrial respiratory chain, accompanied by ATP synthesis. An increase in the glycerol-3-phosphate level at the late stationary stage of growth also confirms it (Figure 7). It is possible that glucose is formed via the conversion of galactose, which shows a high level at the three-week growth stage (Figure 7). The reactions undergo the Leloir metabolic pathway, which includes three enzymes, namely galactokinase (GALK), galactose-1-phosphate uridyl-transferase (GALT), and UDP-galactose-4-epimerase (GALE). This pathway comprises galactose phosphorylation, UDP-galactose synthesis, and its isomerization into UDP-glucose, which can then be used to produce free glucose [65]. An increase in galactose phosphate at the four-week growth stage also supports this assumption (Figure 7).

**Figure 14:**
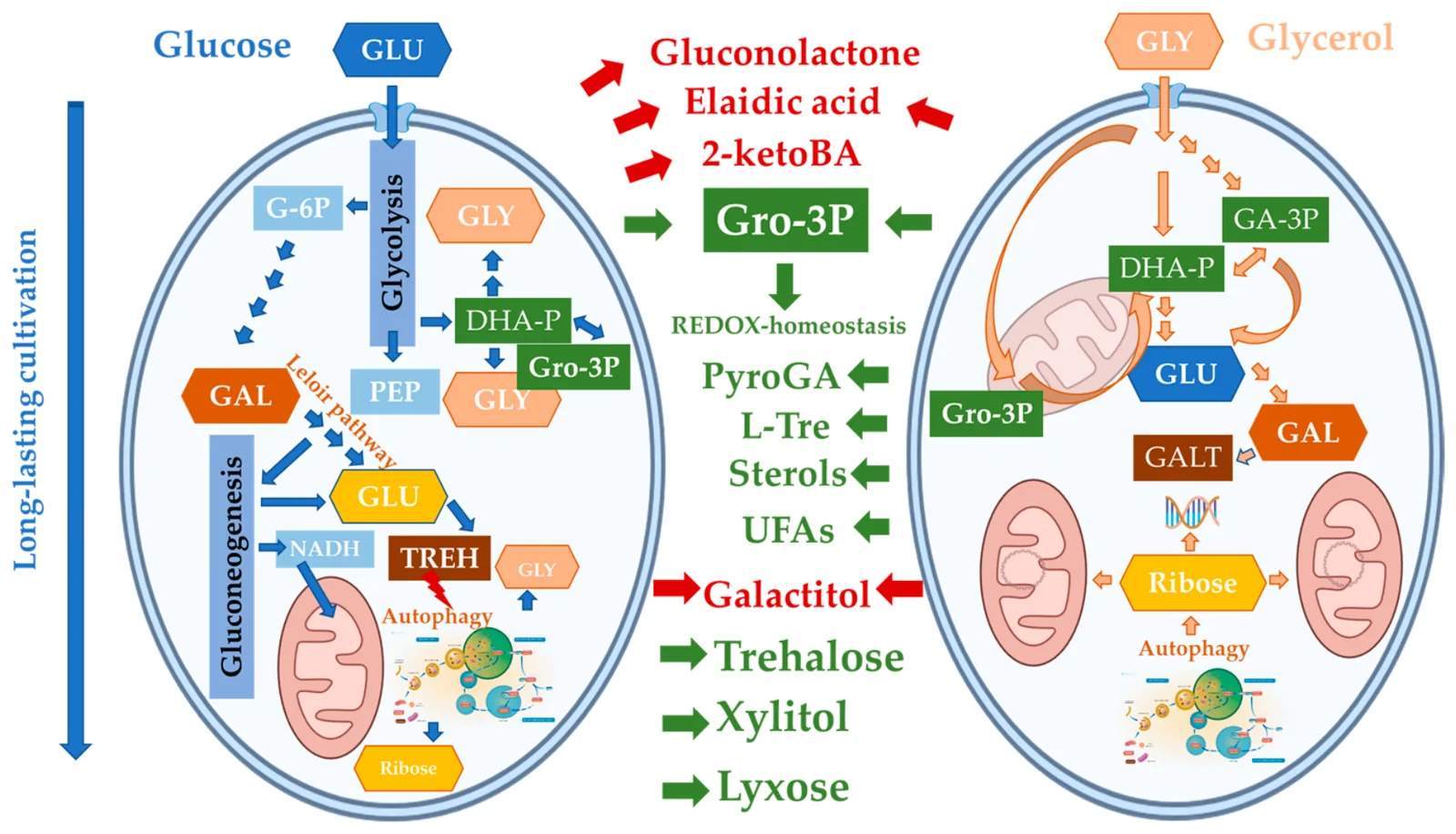
Main metabolic pathways involved in the long-term cultivation of *E. magnusii* yeasts digesting different types of substrates. Metabolic features associated with long-term cultivation are shown in red, while metabolites exerting a protective effect on cellular senescence are shown in green. A detailed description is given in the Section 4. Footnotes: GLU—glucose; GLY—glycerol; G-6P—glucose-6-phosphate; PEP—phosphoenolpyruvate; Gro-3P—glycerol-3-phosphate; GAL—galactose; TREH—trehalose; DHAP—dihydroxyacetone phosphate; GA-3P—glyceraldehyde-3-phosphate; 2-ketoBA—2-ketobutyric acid; PyroGA—pyroglutamic acid; L-Tre—L-threonine; UFAs—unsaturated fatty acids.

The dynamics of glycerol amount in the cells displayed a completely different picture. The polyol level in the glycerol-utilizing yeast, unlike that in the glucose-utilizing yeast, gradually decreased upon cultivation (Figure 4). Obviously, this decrease matched an increase in glucose levels in the first and second-week states (Figure 4). Consequently, at the two-week cultivation stage, a metabolic restructuring happens in the yeast cells followed by a redistribution of the glycerol pool to support the cell energy needs in glucose (Figure 14). In the yeast population grown with glucose as a substrate, glycerol increased in the daily culture, which agrees well with our previous data [37]. The polyol levels also rose at two- and four-week growth stages (Figure 10). A reasonable explanation may be associated with autophagic processes in the cells at the late stationary growth stages. Previously, we stained the cells in vivo with a specific LysoTracker Red DND 99 probe to assay the lysosomal activity of the *E. magnusii* cells under different cultivation conditions [38]. We showed that nearly all the cells cultured with 2% glucose showed high probe fluorescence at the four-week growth stage. The process of autophagy is known to be obligatory upon nutrient depletion to supply the cells with some building blocks from the degraded macromolecules. The importance of autophagy is emphasized by the fact that it is of a strictly regulated nature, where several regulatory components, including TORC1, PKA, Snf1, Sch9, and Rim15, are involved in dynamic autophagic activity [66]. Taking into account the main postulates of the regulation, we can conclude that a decrease in the nutrient availability, on the one hand, triggers TORC1 signals and autophagy, and on the other hand, maintains a relatively high level of ROS, ensuring a high lifespan in the conditions that we demonstrated upon assessing yeast cell viability (Figure 3). An increase in ribose level by the four-week growth stage in the cultures on both substrates, possibly due to the induction of protein synthesis (Figure 7 and Figure 10), also confirms this conclusion.

Thus, our study has shown that glycerol is a key metabolite in the cells of aging yeast culture, which is especially important at the two-week growth stage. We believe that the high glycerol pool upon glucose assimilation at this stage should be related to the cell’s need for the synthesis of its phosphorus derivative, glycerol-3-phosphate (Gro3P). Gro3P biosynthesis is known to restore cytosolic NAD+, releasing mitochondrial pathologies, while the disruption of Gro3P synthesis inhibits yeast proliferation and shortens the lifespan of C. elegans. This confirms that Gro3P biosynthesis is an evolutionarily conserved coordinator of NADH redox homeostasis/NAD+ [67]. Our data show that the dynamics of the Gro3P level in the glucose-assimilating population is completely identical with that of the glycerol amount (Figure 10). Moreover, the intersection of glucose and glycerol metabolism pathways, observed in the study, also occurs in the key Gro3P node, which, being a supplier for dihydroxyacetone-phosphate (DHAP), can coordinate the glycolysis and gluconeogenesis pathways along with de novo glycerol synthesis (Figure 14).

All the stages of yeast growth on both glucose and glycerol contained polyol arabitol (Figure 7 and Figure 10). However, the inositol amount was the highest at the late stationary growth stages, and the trehalose level was significantly higher in the glucose-assimilating yeast compared to those grown on glycerol, which agrees well with our data from [37]. Trehalose accumulation has been described for fungi of *Debaryomyces* and *Geotrichum* [55,68], yeast of *Kluyveromyces lactis* [69], and *Candida albicans* [70] at thermal, oxidative, and osmotic stresses. The protective features of trehalose are believed to be related to its chemical chaperone properties, which affect protein folding via direct protein–trehalose interactions, reducing the destructive effects of the stressors on the cell [71]. However, trehalose plays another important role in inducing autophagy independently of the mTOR pathway. This is established by the study of both relative factors associated with the autophagosome structure, namely LC3-II and Atg5, and the levels of phosphorylation of mTOR and its regulatory proteins [72]. Although the trehalose amount in the yeast cells is relatively low, its increase upon glucose utilization at the three- and four-week growth stage probably induces autophagy, which is confirmed by a jump in the ribose level (Figure 7). Thus, the culture can survive for a long time.

The VIP metabolite of carbohydrate nature in the cells growing on glycerol was galactitol (dulcitol) (Figure 9B), although, according to a comparative assay, the highest amount of the metabolite was observed at the late stationary stages upon glucose utilization (Figure 13A). Galactitol, also known as dulcitol, is a product of the metabolic degradation of galactose and participates in its metabolism. The main metabolism of galactose proceeds through the Leloir pathway; however, some of it can be converted to galactitol with aldose reductase, and then converted to galactonic acid with galactose dehydrogenase [73]. The yeast cells growing on glycerol maintained a high galactose level, as well as galactose phosphate throughout the deep stationary phase, starting from the two- and three-week growth stage (Figure 6B), which indicates that the Leloir pathway maintains carbohydrate balance in the trend of the “respiratory” metabolism under those conditions. On the contrary, upon glucose utilization, the galactose phosphate level reached its maximum by the second week of growth, decreasing to its minimum by the end of the experiment (Figure 9B), which indirectly indicated the redirection of the metabolic flow to the galactitol synthesis (Figure 14). Most likely, it is generated to prevent the intracellular accumulation of galactose-1-phosphate, being a phosphorylated intermediate of the Leloir pathway (Figure 14), which is toxic to the cell [74]. In the *S. cerevisiae* yeast utilizing galactose, galactose-1-phosphate negatively affected the utilization of galactose via inhibition of phosphoglucomutase. Moreover, the authors suggested that the combination of the increased intracellular galactitol level and the ratio between galactose-1-phosphate amount and phosphoglucomutase activity should be important for galactose-related toxicity, which decreases the growth rates in *S. cerevisiae*. In our experiments, galactitol is supposed to play a dual role; on the one hand, it prevents the accumulation of toxic galactose–1-phosphate, and on the other, it acts as an osmolyte in aging cells. A detailed description of its role in the diagnosis of aging signs in the yeast is described in the section “Metabolic signs related to long-term cultivation”.

Thus, based on the results obtained, we could conclude that the induction of gluconeogenesis and Leloir pathways are the main metabolic pathways in the cell carbohydrate metabolism involved in the adaptation of yeast cells to long-term cultivation upon glycerol utilization. Polyols and carbonic acids dominated in the identified compounds. The galactose and galactinol found in the VIP metabolites, formed from the gluconeogenesis reactions of glucose-6-phosphate, are additional arguments in favor of the conclusion. Galactinol may also appear as compensation for osmotic stress due to a high concentration of glycerol or its metabolites.

The predominant classes of monosaccharides and their derivatives, namely polyols and carboxylic acids among the VIP compounds, could indicate that aerobic catabolism and synthesis of reserve carbohydrates are the main metabolic pathways of cell carbohydrate metabolism involved in the adaptation of yeast cells to long-term cultivation upon glucose utilization. Fructose-6-phosphate and galactose-6-phosphate are direct markers of the induction of the glycolytic pathway and the initial stages of glucose oxidation, while the increased amount of citric and fumaric acids shows a high activity of the tricarboxylic acid cycle. However, the appearance of trehalose and dulcitol, as well as galactinol and myoinositol, indicates the synthesis of reserve carbon forms and regulation of osmoprotection.

The accumulation of trehalose at the three- to four-week growth stages is a key tool for maintaining culture survival in the deep stationary phase. In both versions (glycerol and glucose), the compound formed into a cluster, mainly accumulating by the end of cultivation. Trehalose probably acts as an osmoprotector and cryoprotector in our experiments, increasing the stability of cell components upon prolonged growth [75].

### 4.2. Lipid Metabolism: Trans-Unsaturated Fatty Acids as Markers of the Yeast Cell Adaptation upon Long-Term Cultivation

Some studies using yeast models showed that the metabolites of sphingolipids (ceramide, sphingosine, and sphingosine-1-phosphate) actively participate in signal transmission pathways, modulating some processes, including the reactions to stress, aging, and apoptosis. They affect oxidative stress and chronological lifespan [26,76]; however, genetic manipulations on lipid synthesis pathways increased stress sensitivity and reduced CLS [77]. Nevertheless, the treatment of yeast cells with polyunsaturated acids increased ROS generation, which negatively affected respiration and shortened their lifespan [78]. The experiments on lithocholic acid caused some age-related restructuring of the mitochondrial system, confirming the relationship between mitochondrial lipidome and mitochondrial redox status, which indicated the role of lipid metabolism in the lifespan of the yeast population [32].

The studies using the PLS-DA method showed that upon glycerol utilization by yeast cells, some organic acids, namely methylglycerol, palmitic, and linoleic acids (Figure 6B), contributed most to the data clustering, while the caprylic and stearic acids (Figure 9B) did the same in the glucose-assimilating yeast. The results were consistent with our previous data [38]. The synthesis of free fatty acids in yeast cells is known to proceed through three main pathways, namely (1) de novo synthesis; (2) hydrolysis of complicated lipids and protein delipidation; and (3) from external sources [79]. Assimilation of 2% glucose induces de novo synthesis of free fatty acids in the cytosol and mitochondria, followed by elongation and desaturation in the endoplasmic reticulum (EPR). In particular, saturated fatty acid levels upon glucose utilization exceeded those of the unsaturated fatty acids, while growing on glycerol led to high amounts of the polyunsaturated linoleic acid up to the second week of growth; in glucose-assimilating cells, it was not detected at all (Figure 7 and Figure 10). Based on the data, it can be assumed that the cells assimilating glycerol as an “oxidative” substrate have a higher potential for survival compared to the cells growing on glucose.

The VIP metabolites of lipids class in glycerol-growing cells also included palmitylglycerol and sterols (ergosterol and dehydro-ergosterol) (Figure 6B), while in the glucose-growing cells it was only ergosterol (Figure 9B). Previously, we showed that upon long-term cultivation of the *E. magnusii* yeast, the total sterol amount doubled at the three-week growth stage and made up 30% and 62% of the total amount of the lipids at the four-week growth stage for glycerol and glucose, respectively [38]. Sterols are nonpolar lipids protected by sphingolipid heads inside the membranes. Ergosterol, the final product of a complicated pathway of sterol biosynthesis, is embedded in the plasmalemma and keeps its integrity, being the main sterol of the *S. cerevisiae* yeast [80]. Ergosterol, being the most important component of fungal cell membranes, provides the fluidity, permeability, and activity of membrane-bound proteins. Some studies demonstrate that glycerol induces the production of triacylglycerols (TAG) and ergosterol in the *Yarrowia lipolytica* yeast [81]. The identification of ergosterol and its precursor dehydroergosterol among the VIP metabolites (Figure 6, Table 1) shows that the cell membrane needs strengthening. Glycerol, as an osmoprotector, requires restructuring of membrane structures, which leads to activation of the sterol biosynthesis pathway [82]. Therefore, the role of sterols (in particular, ergosterol) and long-chain fatty acids in adapting to stress is probably to stabilize the membranes due to nutrient depletion [83]. Being important components of cell membranes, the metabolites can participate in the compensation of osmotic pressure and toxicity of under-oxidized metabolites. Thus, the accumulation of sterols in the yeast cells upon long-term cultivation could be regarded as a protective factor of adaptation to oxidative stress during aging.

In the matrix of the comprehensive assay of the cell metabolome upon growing on different substrates, at late stages of ontogenesis, the linoleoyl and palmitoyl derivatives of polyol (linoleoylglycerol and palmitoylglycerol) in the glycerol-assimilating yeast appear (Figure 13B). The biosynthesis of linoleoylglycerols and palmitoylglycerol (esters of glycerol and corresponding fatty acids) includes the acylation of glycerol-3-phosphate with fatty acids, converting into the phosphatidic acid, followed by forming diacylglycerols and, finally, triacylglycerols (TAG) with linoleyl residues. The main biosynthetic pathways of the derivatives involve the hydrolysis of more complex lipids (the main pathway)—diacylglycerols (DAG) or TAG [84]. Also, the formation of the metabolites in the membrane lipid rearrangement upon participation of the enzyme phospholipase A1 (PLA1) is quite possible [85]. The presence of palmitoylglycerol glyceride (Figure 6, Table 1) and abundant long-chain fatty acids (palmitic, linoleic, elaidic) among the VIP compounds upon cultivation on glycerol indicates intensive synthesis and modification of membrane lipids. If cultivated on glycerol, yeast most likely switches to the accumulation of reserve lipids (triacylglycerols) in response to stress or osmotic pressure. It is likely that in the late stationary growth stages, a restructuring of the lipid profile associated with the alteration of the membrane apparatus and the hydrolysis of reserve lipids occurs.

### 4.3. Amino Acids: The Role of Branched-Chain Amino Acids in the Yeast Cell Adaptation upon Long-Term Cultivation

A deficiency or excess of amino acids can affect the lifespan of the cells and organisms, but little is known about the role of the level dynamics of free intracellular amino acids in the aging process. In [86], the authors assayed the level of free amino acids upon chronological aging of non-dividing and dividing yeast of *Schizosaccharomyces pombe*, comparing the wild-type cells to the long-lived mutants, which lacked the protein Pka1 of the protein kinase A signaling pathway. In the wild-type cells, the total level of amino acids decreased upon aging, but much less than that in the *pka1* mutants. The level of glutamine decreased, especially in the wild-type cells, while the aspartate level increased, especially in *pka1* mutants, which led to the author’s hypothesis that some amino acids could be biomarkers of aging, and their level upon aging may prolong or shorten the lifespan of the culture. We showed that phenylalanine, leucine, glycine, isoleucine, and valine were the major amino acids in the cultures growing on both substrates (Figure 7 and Figure 10). It is noteworthy that the amount of the most identified amino acids increased at the two-week growth stage, while the dynamics of pyroglutamic acid level (a cyclic derivative of glutamic acid or 5-oxoproline), threonine, and serine significantly depended on the substrate type (Figure 7, Figure 10 and Figure 14). Moreover, upon glucose utilization, a high level of serine was detected at the four-week growth stage. The role of serine in the regulation of lifespan has been shown in some papers. Thus, in the *S. cerevisiae* yeast, the application of *L*-serine to a cell culture increased its lifespan in a dose-dependent way. It could undergo the single-carbon pathway of metabolism, indicating the involvement of the conservative and central metabolic node in the regulation of lifespan in eukaryotes [87]. The authors suggested that the effects of exogenous *L*-serine are most likely related to its catabolism. However, at higher concentrations, the amino acid can participate in non-catabolic processes, in particular, in preventing hyperacidity of the medium. In our experiments, the serine level increased considerably upon cultivation on glucose, especially at the four-week growth stage (Figure 10). Based on our previous data, showing that pronounced autophagy was observed in a four-week-old culture [38], a similar mechanism could be present in the *E. magnusii* cells.

The branched-chain amino acids (leucine, valine, isoleucine), being essential components of our diet, are widely known to play a key physiological role in the regulation of protein synthesis, metabolism, and aging. Their effects depend on the macronutrient ratio and imbalance with other amino acids [88]. The elevated BCAA level promotes the mTORC1 pathway, increasing the level of pro-inflammatory macrophages, and decreasing the number of adipogenic progenitor cells in the white adipose tissue. It leads to metabolic disorders and enhances cell aging in mice [89]. Taking into consideration the omnitude of the TOR pathway regulation in all eukaryotic organisms, we can presume that an increase in BCAA, especially upon cultivation on glucose (Figure 7, Figure 10 and Figure 13C) may probably indicate the aging processes in the yeast population, which is extremely specific depending on the type of substrate. Also, unlike mammals, the *E. magnusii* yeast is capable of its own amino acid synthesis except for methionine, histidine, and tryptophan. Thus, here, we are dealing with damage to the catabolism of some BCAAs due to aging of the cell population.

Besides the main BCAAs significantly contributing to yeast metabolism upon growing on glucose, a high constant level of threonine was found in glycerol-assimilating cells (Figure 7 and Figure 10). Accumulation of threonine, as well as serine, was noted for replicatively aging *S. cerevisiae* cells [90]. Summarizing the data, threonine, being a Krebs cycle-derived amino acid, is supposed to play a key role in respiratory metabolism, maintaining the survival of yeast cells and their lifespan.

The pyroglutamic acid, or 5-oxoproline, also contributed considerably to the metabolism of yeast utilizing glycerol (Figure 6, Figure 9 and Figure 13). 5-oxoproline is a pivotal endogenous intermediate in the *γ*-glutamyl cycle, which is crucially important for glutathione synthesis. It is converted to glutamate by 5-oxoprolinase (OPLAH), thereby replenishing the intracellular pools of reduced glutathione [91]. Recent studies have revealed the regulatory role of 5-oxoproline and OPLAH in maintaining both metabolic and redox homeostasis of the cell [92]. Thus, the increased 5-oxoproline level and OPLAH induction restore the GSH metabolism and probably reduce the oxidative stress associated with aging of the yeast culture, maintaining its high antioxidant potential and preventing cell death. This opinion is supported by the fact that we observed a jump in the 5-oxoproline level at the two-week growth stage upon glycerol utilization (Figure 6), when, possibly, there is restructuring of the culture metabolic profile and an adaptive response to oxidative stress develops. Along with this, upon glucose metabolism, on the contrary, a sharp drop in the amino acid level occurs in the four-week-old culture (Figure 9 and Figure 13C).

Thus, amino acid metabolism clearly differentiates the carbon metabolism specificity, reflecting the difference in protein synthesis and nitrogen metabolism. Glycerol stimulates the accumulation of pyroglutamic acid, threonine, and valine, while glucose promotes the accumulation of phenylalanine, leucine, isoleucine, and serine. Glycine is a universal marker that may indicate its role in universal metabolic pathways (for example, in the synthesis of glutathione or purines).

### 4.4. Metabolic Signs Related to Long-Term Cultivation

Metabolomics provides unique, organized approaches to the study and comprehension of the aging process, as it allows us to get an objective and full profile of metabolites formed due to the physiological response of a cell to different internal and external factors [11]. Some recent studies have established a close relationship between aging and nutrient uptake, lipid, carbohydrate, and amino acid metabolism, and redox homeostasis. Since metabolic reactions can lead to the accumulation of by-products of hurting effect on cells, a thorough study of the manifestation type, frequency, and intensity is needed. Moreover, to establish a link between them and the aging process, the features of the accumulated damage should be revealed [11]. Thus, the application of a non-targeted approach to profiling the metabolome of the yeast population as a model of unicellular eukaryotes at different age stages could provide a profile of metabolites being possible metabolites associated with adaptation upon long-term cultivation. Having analyzed the metabolic profiles, we made some conclusions: (1) metabolic signatures associated with long-term cultivation/chronological aging was most clearly displayed in the glucose-assimilating cells; (2) the candidate metabolic markers of aging were identified in all the classes of compounds tested, which allows us to use the metabolites as the aging signatures for other objects, including higher eukaryotes; (3) most metabolites, being the metabolic signs of aging, are at the same time the protective agents (Figure 14).

#### 4.4.1. Gluconolactone

In glucose-assimilating cells, gluconic acid (in the form of lactone) reached rather high levels by the fourth week of cultivation (Figure 13B). In [93], lactones were used as the indicators of aging in wine, and the authors showed five lactones, which positively correlated with age in the Madeira wines, namely pantolactone, *γ*-ethoxybutyrolactone, *γ*-heptalactone, trans-oak lactone, and cis-oak lactone (pantolactone, *γ*-ethoxybutyrolactone, *γ*-heptalactone, trans-oak lactone, and cis-oak lactone). Some studies have shown that gluconolactone formed in the pentose phosphate pathway can be used as a marker of some mammalian pathologies [94]. It is of note that the protein Senescence Marker Protein-30 (SMP30), identified as gluconolactonase (GNL), which is involved in the conversion of gluconolactone into gluconic acid in the biosynthetic pathway of vitamin C, is one of the most important markers of aging in mammals [95]. In a recent study, Dong et al. showed that SMP30 (MrSMP30-1) from the entomopathogenic fungus *Metarhizium robertsii* is needed for ROS elimination and mitochondrial function. Phylogenetic analysis showed that MrSMP30-1 is related more closely to SMP30 proteins of phytopathogenic fungi than to those of other entomopathogens. The removal of MrSMP30-1 led to the accumulation of intracellular ROS, a significant decrease in the number of lipid droplets, and the collapse of the mitochondrial membrane potential, which resulted in accelerated spontaneous degeneration of the fungus. This not only expanded the understanding of the SMP30 role in fungi but also proved that gluconolactonase can serve as a biomarker for monitoring the degeneration of fungal cultures. Thus, gluconolactone and its metabolism in the yeast cells can serve as an important marker of pathology and adaptation to oxidative stress, which is highly conserved in different types of organisms (Figure 14).

#### 4.4.2. Galactitol (Dulcitol)

Upon glucose utilization in the yeast cells, galactitol has also been identified as the most important metabolic marker upon long-term cultivation (Figure 13B). Galactitol, a downstream metabolite of galactose metabolism, is formed due to the galactose hydrogenation through its reduction with aldose reductase, at the energy expense of NADPH [96]. Galactitol is poorly metabolized and cannot diffuse through the cell membranes because of its low liposolubility increasing intracellular osmotic pressure and hurting the membrane [97]. Galactitol has also been proposed as one of the links between excessive galactose effects and cell aging [97,98]. Thus, galactitol was concluded to play a central role in a wide range of cellular disorders and in galactose toxicity in the *S. cerevisiae* yeast [89]. The reasons for the effects may be different aspects of carbohydrate metabolism. On the one hand, galactitol interacts with Ca^2+^ in the cell [99], and it may either negatively affect the enzymes using the cation, or its signaling. On the other hand, the effect of galactitol may be displayed in its production in response to osmotic stress [100] and, therefore, polyol can be an osmo-regulator alternative to glycerol. A possible explanation for the galactitol toxicity may be the synergy of polyol with other metabolites, namely galactose and galactose-P, as it was shown in animal models [101]. As our data show, galactose and galactose-P accumulated in the glycerol-assimilating yeast at the late stationary growth stages (Figure 7); however, it was incomparable to that in the glucose-assimilating yeast (Figure 10 and Figure 13B).

Thus, galactitol, as a metabolic biomarker upon long-term growth of the *E. magnusii* yeast, can exert its effects via all of the proposed mechanisms. It should also be emphasized that the yeast strain we studied may be a successful eukaryotic model for assessing metabolic dynamics related to aging in other eukaryotic organisms (Figure 14).

#### 4.4.3. Elaidic Acid

Elaidic acid was also an important element of the lipidome in the yeast cells on both growth substrates (Figure 6B, Figure 9B, Figure 12, Figure 13B and Figure 14). Elaidic acid (trans-9-octadecenic acid) belongs to the class of monounsaturated fatty acids, being a trans isomer of oleic acid. Monounsaturated fatty acids make up approximately 70% of the total amount of intracellular fatty acids in *S. cerevisiae* cells grown on glucose, and are mainly cis-unsaturated fatty acids with 16 or 18 carbon atoms [90]. The compounds are synthesized from saturated acyl-CoA precursors via desaturation with Δ9, Ole1 desaturase [102]. Its expression strongly depends on growth conditions, in particular, oxygen level and temperature [103,104]. The role of elaidic acid in yeast physiology was studied using a strain of *S. cerevisiae* [105]. The authors compared the physiological effects of both Δ9 cis and trans 18-carbon monoenic fatty acids (the oleic and elaidic acids) in the yeast cells and showed that the elaidic acid, unlike oleic acid, reduced the accumulation of neutral lipids in wild-type yeast. Taking into account the structure of the elaidic acid, it has been suggested that the elaidic acid may act similarly to the saturated fatty acids in the yeast cells. In [106], it was shown that some trans-unsaturated fatty acids, including the elaidic acid (a trans-isomer of oleate), were highly effective in suppressing autophagy and endoplasmic reticulum stress caused by palmitic acid. The elaidic acid also inhibited the autophagy induction with palmitic acid in vivo in the liver and heart of mice, which led the authors to conclude that this fatty acid can reverse cytoprotective stress reactions due to the saturated fatty acids. In our experiments, the elaidic acid level increased sharply in glucose-assimilating yeast at the two-week growth stage, while in glycerol-growing yeast, this metabolite was detected only at the three- and four-week growth stages in significantly low amounts (Figure 7, Figure 10, Figure 12, Figure 13B and Figure 14). We can assume that the trans-monounsaturated acid acts as a kind of metabolic signal at some special points in the yeast cultivation process.

#### 4.4.4. 2-Ketobutyric Acid

2-ketobutyric acid was considered another important metabolic marker in the senescent *E. magnusii* cells upon growing on glucose (Figure 12B and Figure 13). For the yeast, some ways of synthesizing 2-ketobutyric acid have been described, namely, 1) either from *L*-homoserine with the activity of the cystathionine-γ-lyase (CYS3), which catalyzes the conversion of cystathionine to 2-ketobutyrate [107] or from O-acetyl-*L*-homoserine in the reaction of cystathionine-γ-synthase (YML082W) [108]; 2) from threonine upon the action either of threonine deaminase encoded by the *CHA1* gene, or the threonine ammonolysis encoded by the *ILV1* gene; 3) from pyruvate through the metabolic pathways associated with carbon assimilation, often involving propionyl-CoA [109]. The role of ketobutyrate in the regulation of lifespan has been studied using animal models. Thus, the application of the α-ketobutyrate (α-KB) can prolong life expectancy [110] in mice and stimulate autophagy [111]. Based on both our data and on some authors’ studies based on animal models, it can be assumed that α-KB may be a possible metabolic marker, being, in its turn, an agent which stimulates autophagy and promotes the survival of the yeast population upon long-term cultivation (Figure 14).

## 5. Conclusions

Yeast cells are an excellent tool for studying cell and organismic aging at the level of a single cell, and the *E. magnusii* species, being an obligate-aerobic strain, showed pronounced oxidative metabolism with a fully competent mitochondrial respiratory chain similar to that of higher animals. This provides the application of metabolic technologies to the discovery of age-related biomarkers and age-related morbidity.

Our results showed that *E. magnusii* demonstrates an extremely high survival rate and energetic activity in the deep stationary growth phase (3–4 weeks), which is accompanied by an active restructuring of the metabolome. Unlike most yeast species, where metabolism expires in the stationary phase, the *E. magnusii* cells accumulate some specific metabolites at a late stationary growth stage, which is confirmed by clustering of the samples in the PCA and PLS-DA models. The identified palmitoylglycerol glyceride (Figure 6, Table 1) and a lot of long-chain fatty acids (palmitic, linoleic, and elaidic) among the VIP metabolites when cultured on glycerol indicate intensive synthesis and modification of membrane lipids. When cultivated on glycerol, the yeast culture most likely switches to the accumulation of reserve lipids (triacylglycerols) in response to stress or osmotic pressure.

Based on our results, we can conclude that the induction of lipid metabolism and gluconeogenesis pathways are the main metabolic pathways involved in the adaptation of yeast cells to long-term cultivation upon glycerol utilization, whereas if growing on glucose, the functions are performed by aerobic catabolism and synthesis of reserve carbohydrates. This is confirmed by the presence of the predominant classes of monosaccharides and their derivatives, polyols, and carboxylic acids among the VIP metabolites.

The accumulation of trehalose, ergosterol, and its derivatives at three to four weeks of growth is a key mechanism for maintaining culture survival in the deep stationary phase. In both variants (glycerol and glucose), the compounds enter a cluster, accumulating by the end of the experiment. Trehalose acts as an osmoprotector and cryoprotector, while ergosterol increases the stability of cell membranes.

Also, some key metabolites probably formed upon culturing on glycerol (palmitic, linoleic, and malic acids) and accumulated during the transition period (2–3 weeks). The organic acids can act not only as metabolites, but also as signaling molecules, which trigger transcription factors regulating the stress response (for example, the Ras-cAMP or TOR pathway). It initiates the transition of cells into a long-lived state. And thus, glycerol contributes to a high survival rate of *E. magnusii* due to the early launch of adaptive oxidative stress, the active use of lipids as an energy source, and the accumulation of membrane sterols and osmo-protective polyols. Glycerol also induces moderate oxidative stress, which is not damaging, but hormetic (adaptive). It stimulates the activity of antioxidant systems (CAT, SOD, glutathione), increases the resistance of the population to subsequent damage, which creates a state of “controlled stress” in the cells, which increases the whole resistance and maintains high survival for four weeks.

By contrast, growth on glucose provokes a sharp depletion of the substrate and a shock switch to “oxidative” carbon sources, forcing the cells to rely on passive storage of sugars (in particular, trehalose), which provides only lower survival. Also, in this case, antioxidant activity increases only with age, which leads to later and less effective protection.

Thus, the high potential for cell survival upon cultivation on glycerol is not a passive consequence of depletion, but an active, genetically controlled metabolic transition trending toward conserving resources and structural protection. *E. magnusii* demonstrates deep metabolic plasticity, completely rearranging its pool of polyols, sugars, organic acids, and lipids depending on the carbon source. The OPLS-DA model proves that the dependence on the substrate type is systemic and dominates over some temporary changes. A set of specific metabolites (VIP > 1.5), especially elaidic acid, 2-ketoisovaleric acid, and maltose, can serve as reference markers to distinguish the type of metabolism in the yeast species. Moreover, some metabolites, namely gluconolactone, dulcitol, and 2-ketobutyric acid, may be used as specific markers determining adaptive metabolic restructuring upon long-term cultivation.

Analysis of the results of the metabolic profiles led to some conclusions: (1) metabolic signs associated with long-term cultivation were observed in metabolites of all the classes of compounds (polyols, fatty acids, lactones) which may have high potential for use as the indicators of aging for other objects; (2) some metabolites which are hypothetical biomarkers of aging may at the same time be protective agents involved in the adaptation of yeast cells to the deep stationary growth stage. The results obtained can serve as a basis for comparative studies of aging-related metabolism in other eukaryotic models.

Further research involves the validation of the hypotheses presented. In particular, possible paths in this direction may include: (1) inhibition of ergosterol synthesis and assay of lifespan in the stationary phase; (2) introduction of exogenous trehalose into the medium at late growth stages; (3) genetic manipulations with the enzymes determining the key metabolites; (4) targeted metabolomics methods, which allow tracing of the dynamics of important markers in different cultivation conditions.

## Figures and Tables

**Table 1:** Comparative features of the VIP-metabolite classes identified upon yeast cultivation on glycerol and glucose.

Classes	Glycerol	Glucose
Glycerides	Palmitoylglycerol	-
Sterols	Ergosterol * Dehydroergosterol	Ergosterol
Monosaccharides	Lyxose Galactose	Lyxose Ribose Trehalose
Phospho-sugars	Galactose-P	Galactoso-P Fructose-P
Polyols	Galactinol	Galactinol Dulcitol
Carboxylic Acids	Malic acid Benzoic acid Methylglyceric acid Palmitic acid Linoleic acid Elaidic acid	Citric acid Fumaric acid 3-hydroxypropionic acid
Amino Acids	Leucine	Glycine
Phenols	Anthiaergestan	-
Carboxylic Esters	-	Landalol

* Compounds found in both variants are marked in red.

## Data Availability

The original contributions presented in this study are included in the article/Supplementary Materials. Further inquiries can be directed to the corresponding author.

## Supplementary Materials

The following supporting information can be downloaded at https://www.mdpi.com/article/10.3390/jof12080592/s1, Figure S1. The pH dynamics in the cultural medium volume upon the long-term cultivation of *Endomyces magnusii* yeast; Table S1. The amount of the substrates in the cultural medium upon the growth of the *Endomyces magnusii* culture; Figure S2. The level of growth substrates (glycerol and glucose) in the cultural medium upon the long-term cultivation of *Endomyces magnusii*. Footnotes: gly- 0h, glu- 0h; the initial level in the cultural medium; gly 1d, glu 1d; the 24 -h growth stage; gly 1w, glu 1w the one-week growth stage; Table S2: Semi-quantitative analysis of identified metabolites; Table S3: Matrix with the information on the analyses and metabolite identification.

## Abbreviations

The following abbreviations are used in this manuscript:

AMDIS	Automated Mass Spectral Deconvolution and Identification System
BCAAs	branched-chain amino acids
BSTFA	N,O-bis—3-methyl-silyl-3-F-acetamide
DAG	diacylglycerols
EPR	endoplasmic reticulum
GALE	UDP-galactose-4-epimerase
GALK	galactokinase
GALT	galactose-1-phosphate uridyl-transferase
GC-MS	gas chromatography—mass spectrometry
GMD	Golm Metabolome Database
Gro3P	glycerol-3-phosphate
KNN	k-nearest neighbors
NIST	National Institute of Standards and Technology
OPLAH	5-oxoprolinase
OPLS-DA	Orthogonal Projections to Latent Structures Discriminant Analysis
PCA	principal component analysis
PCs	principal components
PI	Propidium iodide
PKCe	protein kinase C
PLS-DA	partial least squares discriminant analysis
RI	retention index
ROS	reactive oxygen species
SeMeCo	self-establishing metabolically cooperating communities
SOD	superoxide dismutase
TAG	triacylglycerols
VIP	variable importance in the projection
α-KB	α-ketobutyrate
