# Supplementary material for: Metabolomic Profiling of Endomyces magnusii During Long-Term Cultivation on Glycerol and Glucose
Source: J Fungi (Basel). 2026 Aug 10;12(8):592. doi: 10.3390/jof12080592 (PMC13514697; doi:10.3390/jof12080592)

Отчёт анализа

Паспорт хроматограммы

Оператор:	Tereshina
Хроматограмма:	№1499 2026-07-01 14:59:28
Файл:	C:\Analytic 3\Projects\Сахара\.chromatograms\2026-07-01 14-59-28 1499.chrx
Хроматограф:	Хроматэк-Кристалл 5000 №: 2251145 Версия прошивки: v 03.21.17.743
Метод:	C:\Analytic 3\Projects\Сахара\.chromatograms\2026-07-01 14-28-02 1498.chrx
Проба:	1a-EM2024 re
Объём пробы:	1
Разведение:	1
Колонка:	ZB-5 30m*0,32mm
Комментарии:	

Результат анализа

Компонент	Время (мин)	Площадь (мВ*с)	Высота (мВ)
Glycerol	3.630	534.698	217.624
IS	11.084	1228.209	480.802

Хроматограмма

ПИД-2

Интервал от 0.000 мин до 26.930 мин

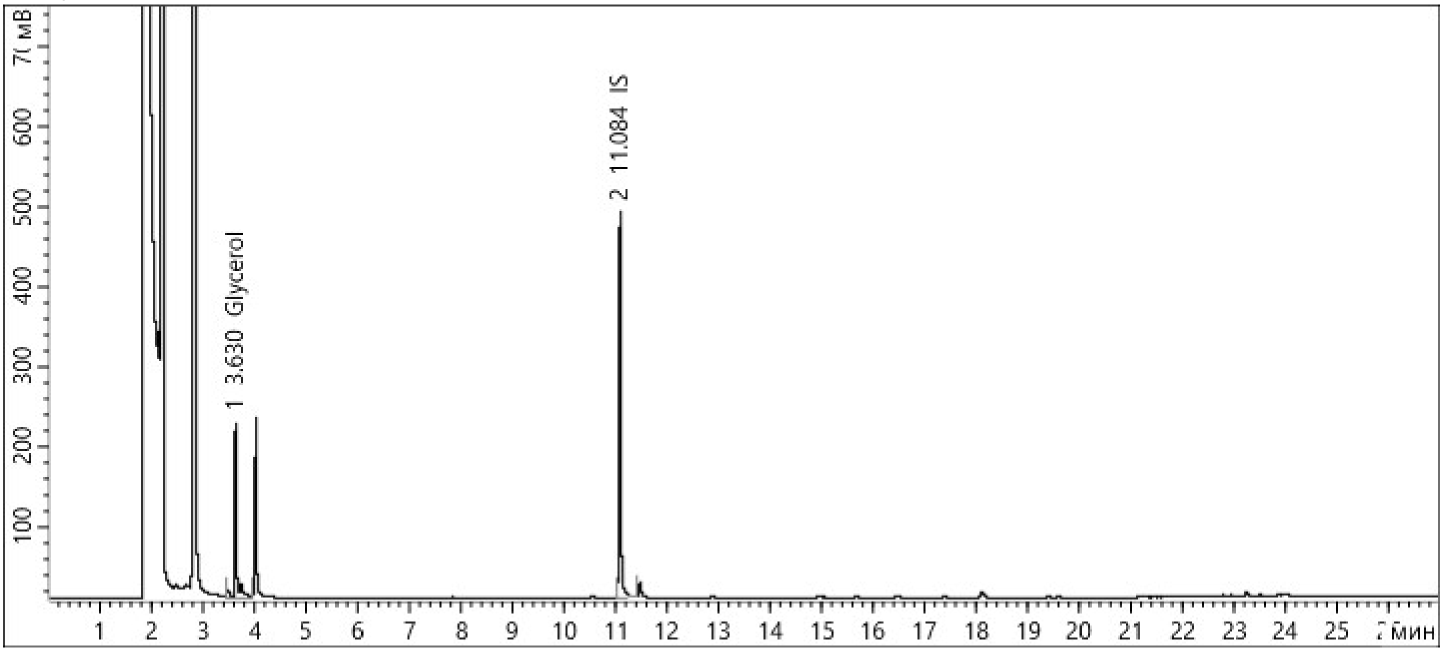

Отчёт анализа

Паспорт хроматограммы

Оператор:	Tereshina
Хроматограмма:	№1495 2026-07-01 12:50:36
Файл:	C:\Analytic 3\Projects\Сахара\.chromatograms\2026-07-01 12-50-36 1495.chrx
Хроматограф:	Хроматэк-Кристалл 5000 №: 2251145 Версия прошивки: v 03.21.17.743
Метод:	C:\Analytic 3\Projects\Сахара\.chromatograms\2026-07-01 12-18-32 1494.chrx
Проба:	2-EM2024 re
Объём пробы:	1
Разведение:	1
Колонка:	ZB-5 30m*0,32mm
Комментарии:	

Результат анализа

**Компонент**	**Время (мин)**	**Площадь (мВ*с)**	**Высота (мВ)**
IS	11.104	3441.049	1300.639
A glucose	12.687	1465.119	609.702
B glucose	14.079	1985.294	688.422

Хроматограмма

ПИД-2

Интервал от 0.000 мин до 26.955 мин

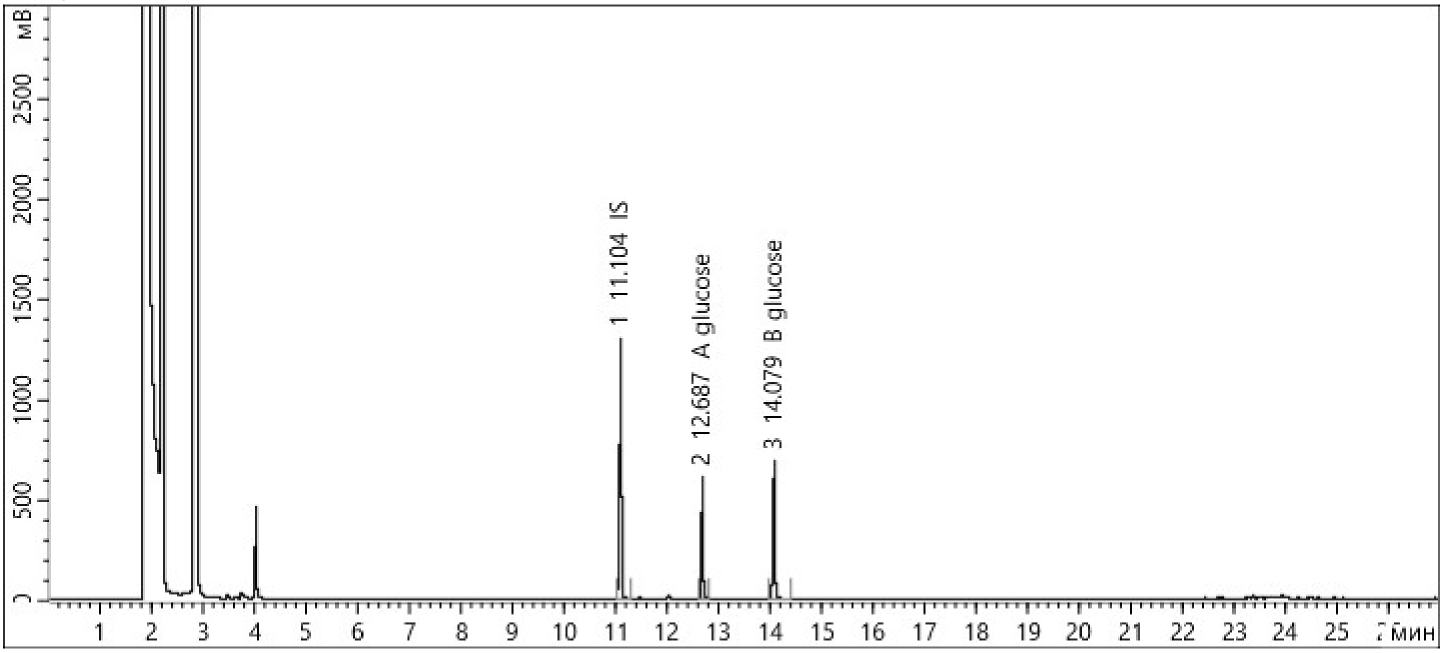

Отчёт анализа

Паспорт хроматограммы

Оператор:	Tereshina
Хроматограмма:	№1497 2026-07-01 13:56:43
Файл:	C:\Analytic 3\Projects\Caxapa\.chromatograms\2026-07-01 13-56-43 1497.chrx
Хроматограф:	Хроматэк-Кристалл 5000 №: 2251145 Версия прошивки: v 03.21.17.743
Метод:	C:\Analytic 3\Projects\Caxapa\.chromatograms\2026-07-01 13-25-27 1496.chrx
Проба:	3-EM2024 re
Объём пробы:	1
Разведение:	1
Колонка:	ZB-5 30m*0,32mm
Комментарии:	

Результат анализа

**Компонент**	**Время (мин)**	**Площадь (мВ*с)**	**Высота (мВ)**
Glycerol	3.640	981.390	640.664
IS	11.129	4922.508	1653.830

Хроматограмма

ПИД-2

Интервал от 0.000 мин до 26.955 мин

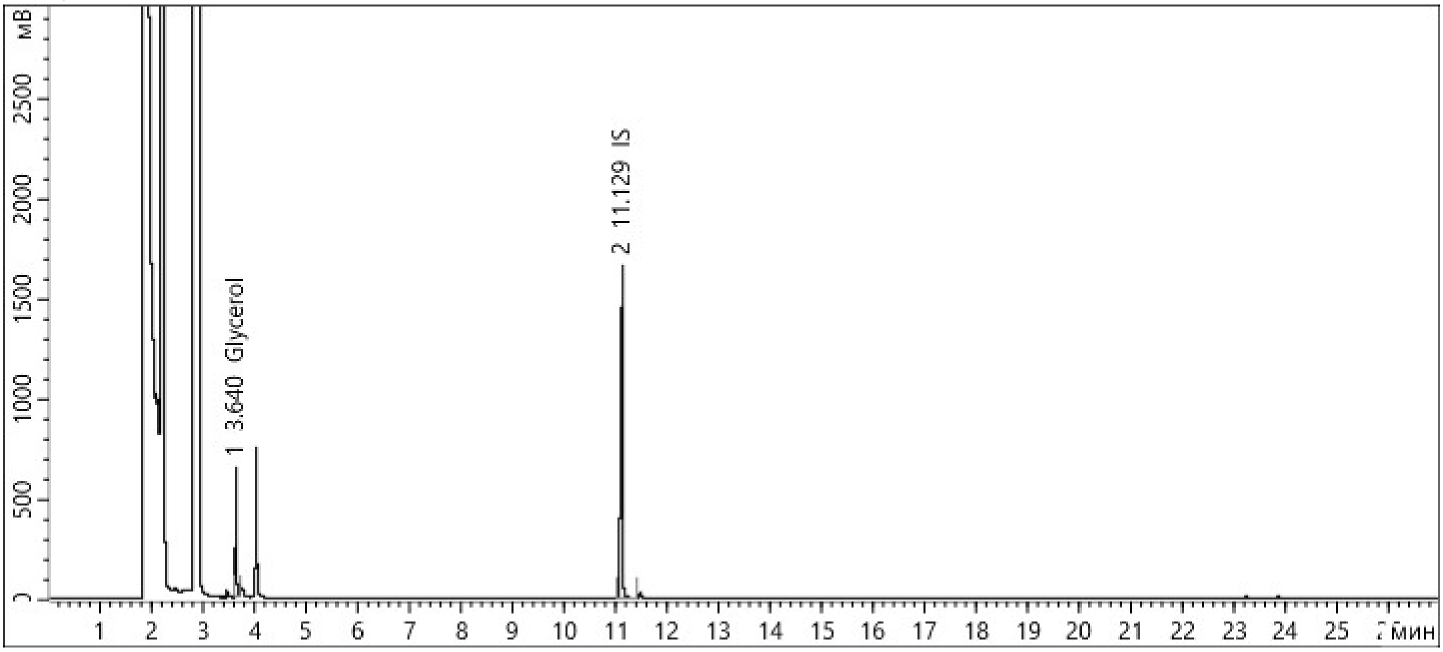

Отчёт анализа

Паспорт хроматограммы

Оператор:	Tereshina
Хроматограмма:	№1500 2026-07-01 15:30:41
Файл:	C:\Analytic 3\Projects\Сахара\.chromatograms\2026-07-01 15-30-41 1500.chrx
Хроматограф:	Хроматэк-Кристалл 5000 №: 2251145 Версия прошивки: v 03.21.17.743
Метод:	C:\Analytic 3\Projects\Сахара\.chromatograms\2026-07-01 14-59-28 1499.chrx
Проба:	4-ЕМ2024 re
Объём пробы:	1
Разведение:	1
Колонка:	ZB-5 30m*0,32mm
Комментарии:	

Результат анализа

**Компонент**	**Время (мин)**	**Площадь (мВ*с)**	**Высота (мВ)**
IS	11.107	3704.222	1365.050
A glucose	12.696	3.583	0.889
B glucose	14.121	5.851	1.221

Хроматограмма

ПИД-2

Интервал от 0.000 мин до 26.951 мин

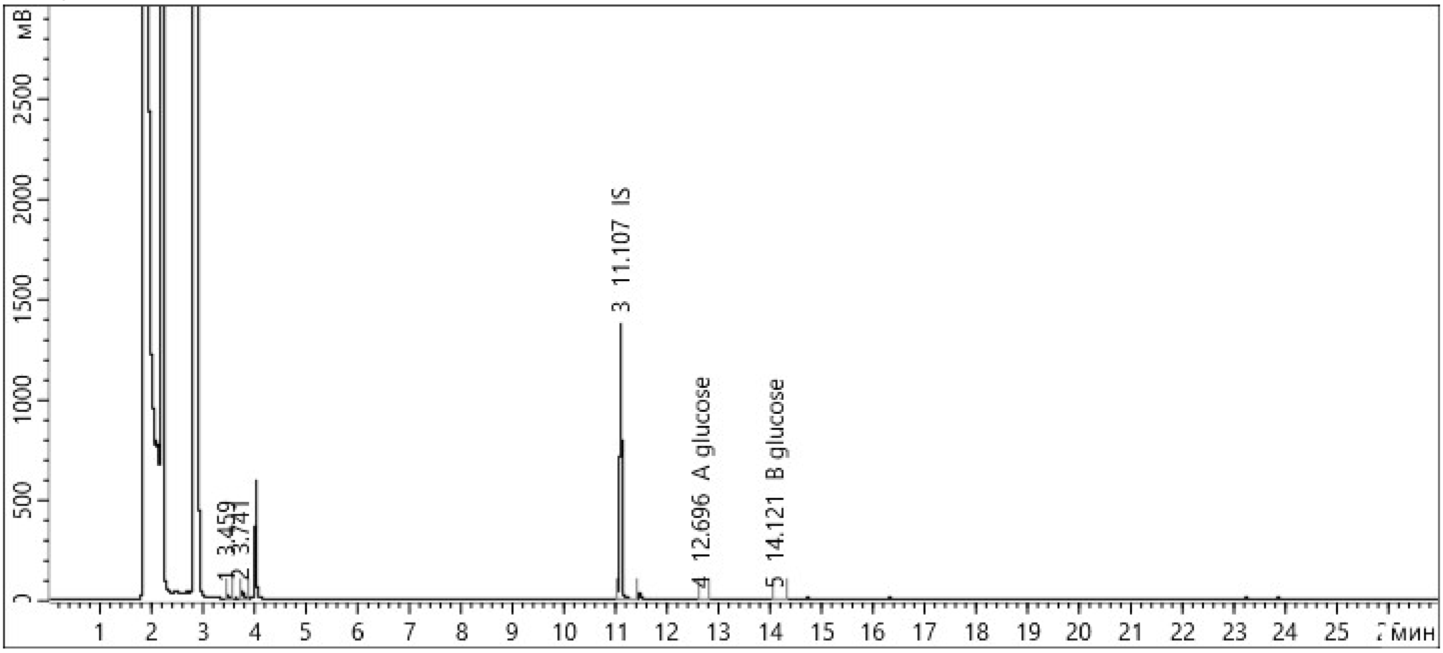

Отчёт анализа

Паспорт хроматограммы

Оператор:	Tereshina
Хроматограмма:	№1503 2026-07-02 10:22:20
Файл:	C:\Analytic 3\Projects\Сахара\.chromatograms\2026-07-02 10-22-20 1503.chrx
Хроматограф:	Хроматэк-Кристалл 5000 №: 2251145 Версия прошивки: v 03.21.17.743
Метод:	C:\Analytic 3\Projects\Сахара\.chromatograms\2026-07-01 16-34-10 1502.chrx
Проба:	5-EM2024 re
Объём пробы:	1
Разведение:	1
Колонка:	ZB-5 30m*0,32mm
Комментарии:	

Результат анализа

Компонент	Время (мин)	Площадь (мВ*с)	Высота (мВ)
Glycerol	3.631	35.243	18.803
IS	11.103	3600.269	1336.915

Хроматограмма

ПИД-2

Интервал от 0.000 мин до 26.935 мин

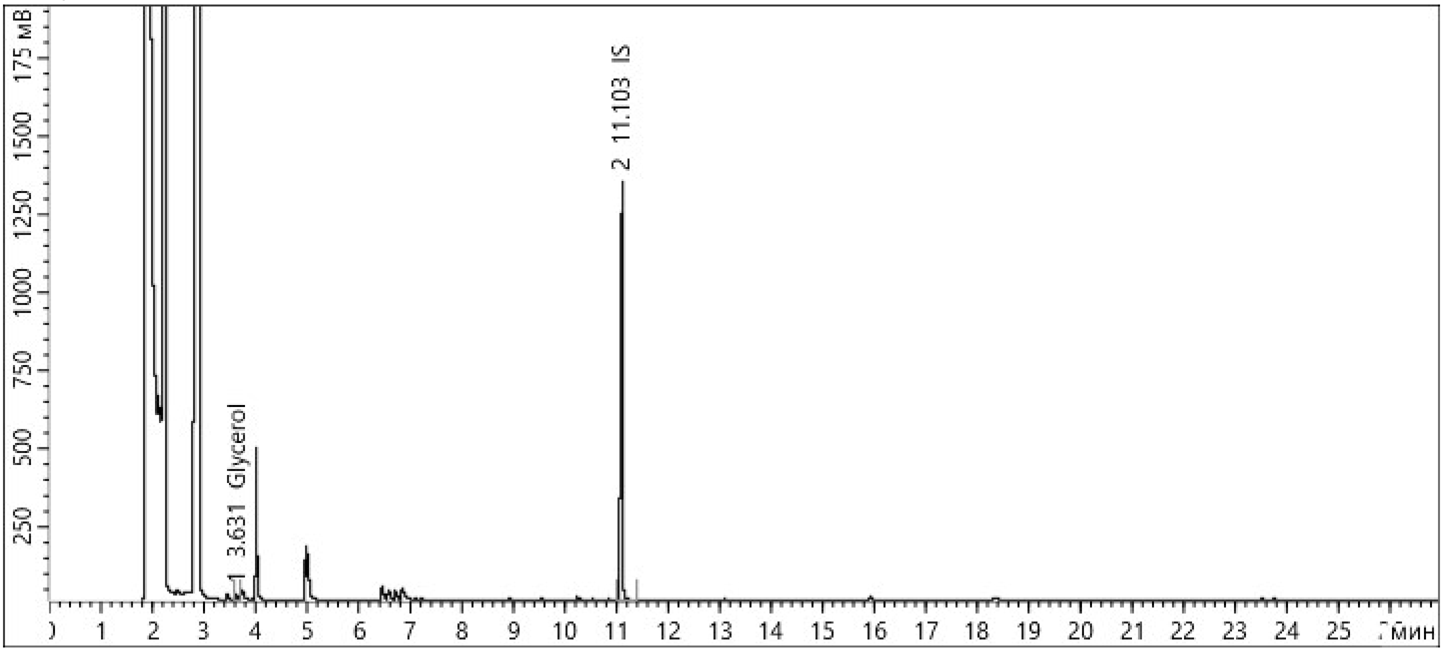

Отчёт анализа

Паспорт хроматограммы

Оператор:	Tereshina
Хроматограмма:	№1506 2026-07-02 11:57:04
Файл:	C:\Analytic 3\Projects\Сахара\.chromatograms\2026-07-02 11-57-04 1506.chrx
Хроматограф:	Хроматэк-Кристалл 5000 №: 2251145 Версия прошивки: v 03.21.17.743
Метод:	C:\Analytic 3\Projects\Сахара\.chromatograms\2026-07-02 11-25-05 1505.chrx
Проба:	6а-EM2024 re
Объём пробы:	1
Разведение:	1
Колонка:	ZB-5 30m*0,32mm
Комментарии:	

Результат анализа

Компонент	Время (мин)	Площадь (мВ*с)	Высота (мВ)
Glycerol	3.638	327.772	187.981
IS	11.076	1553.786	635.915

Хроматограмма

ПИД-2

Интервал от 0.000 мин до 26.931 мин

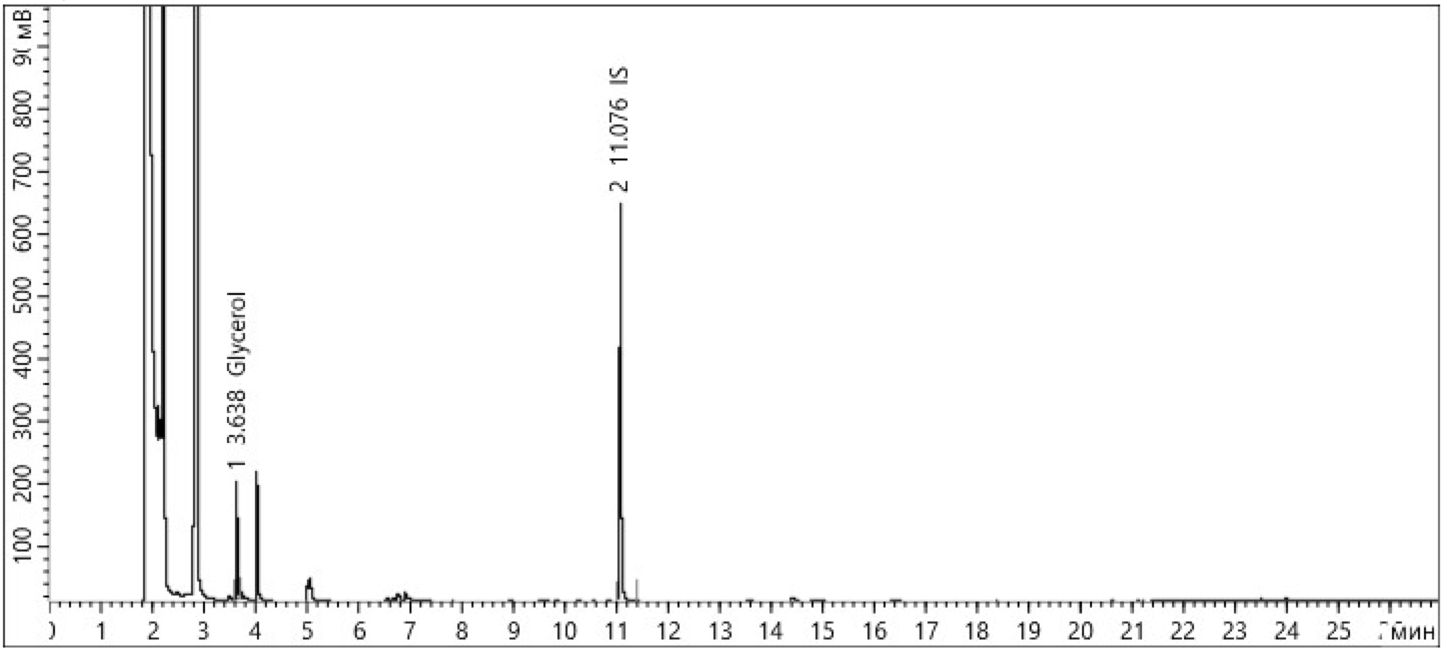

Отчёт анализа

Паспорт хроматограммы

Оператор:	Tereshina
Хроматограмма:	№1508 2026-07-02 13:02:33
Файл:	C:\Analytic 3\Projects\Сахара\.chromatograms\2026-07-02 13-02-33 1508.chrx
Хроматограф:	Хроматэк-Кристалл 5000 №: 2251145 Версия прошивки: v 03.21.17.743
Метод:	C:\Analytic 3\Projects\Сахара\.chromatograms\2026-07-02 12-28-22 1507.chrx
Проба:	Standard mixture (a)-EM2024 re
Объём пробы:	1
Разведение:	1
Колонка:	ZB-5 30m*0,32mm
Комментарии:	

Результат анализа

**Компонент**	**Время (мин)**	**Площадь (мВ*с)**	**Высота (мВ)**
Glycerol	3.636	1803.078	1019.951
Internal stardard	11.069	1518.746	675.557
A glucose	12.660	635.015	256.154
B glucose	14.049	836.372	283.005

Хроматограмма

ПИД-2

Интервал от 0.000 мин до 26.937 мин

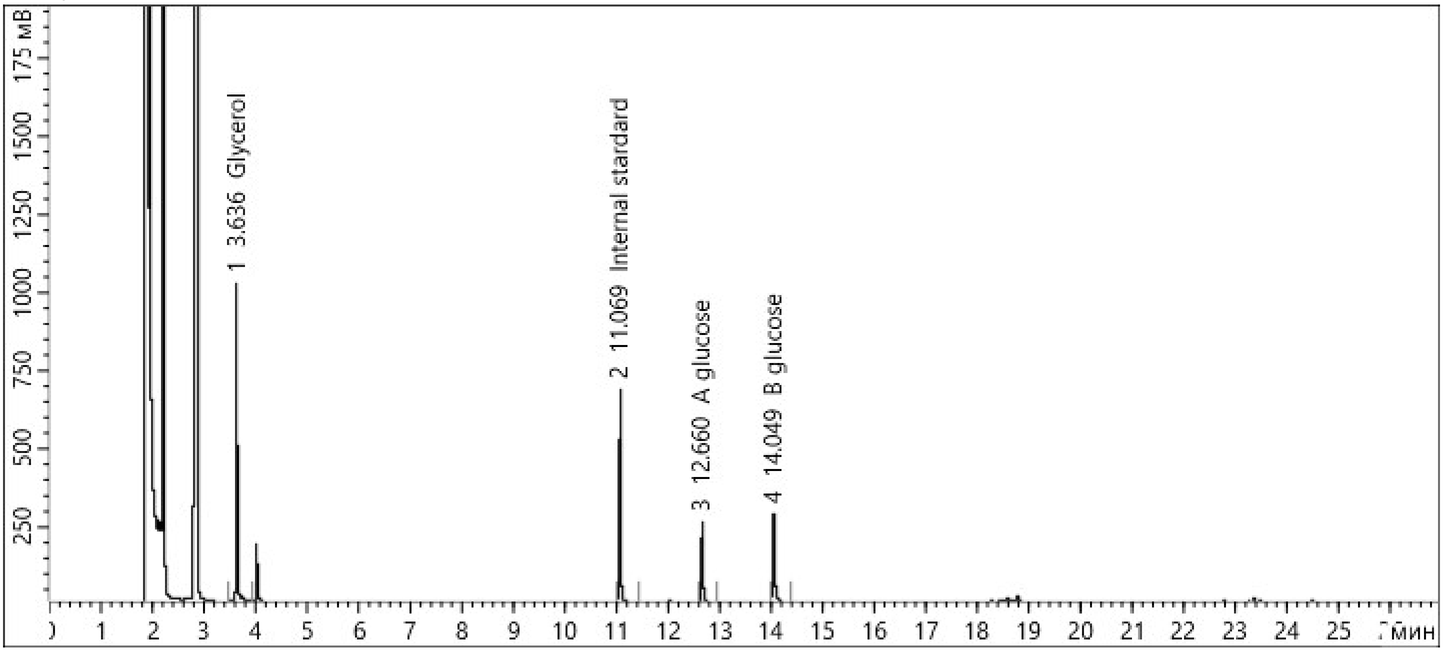

Supplement: Supplementary file 1 [file jof-12-00592-s001.zip › Chromatogramms_united.pdf]
