# Supplementary material for: Metabolomic Profiling of Endomyces magnusii During Long-Term Cultivation on Glycerol and Glucose
Source: J Fungi (Basel). 2026 Aug 10;12(8):592. doi: 10.3390/jof12080592 (PMC13514697; doi:10.3390/jof12080592)
Supplement: Supplementary file 1 [file jof-12-00592-s001.zip › Supporting materials.pdf]

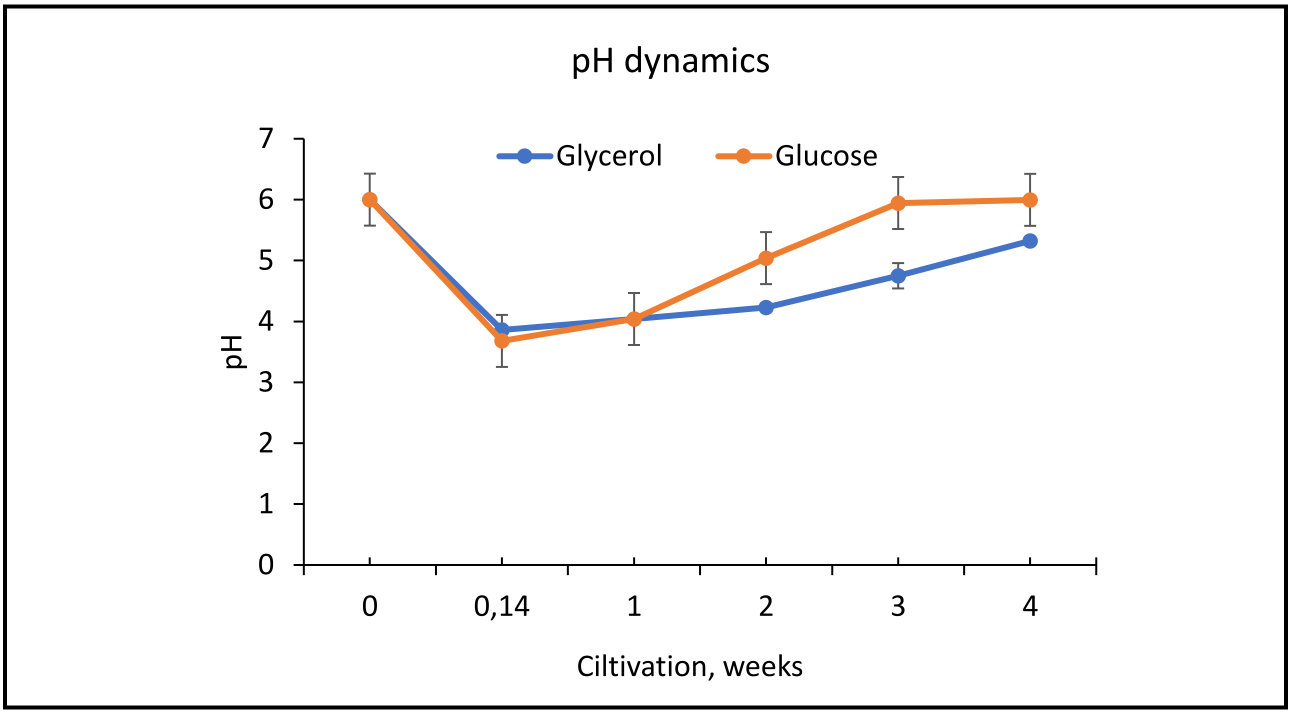

Figure S1. The pH dynamics in the cultural medium volume upon the long-term cultivation of *Endomyces magnusii* yeast.

Table S1. The amount of the substrates in the cultural medium upon the growth of the *Endomyces magnusii* culture

CHs		gly 0h	glu 0h	gly 1d	glu 1d	gly 1w	glu 1w
g/ 100 ml of cultural medium (%)	Glycerol	0,955±0,007071068	0,00	0,31±0,014142136	0,00	0,00	0,00
	Glucose	0,00	0,915±0,06364	0,00	0,00	0,00	0,00

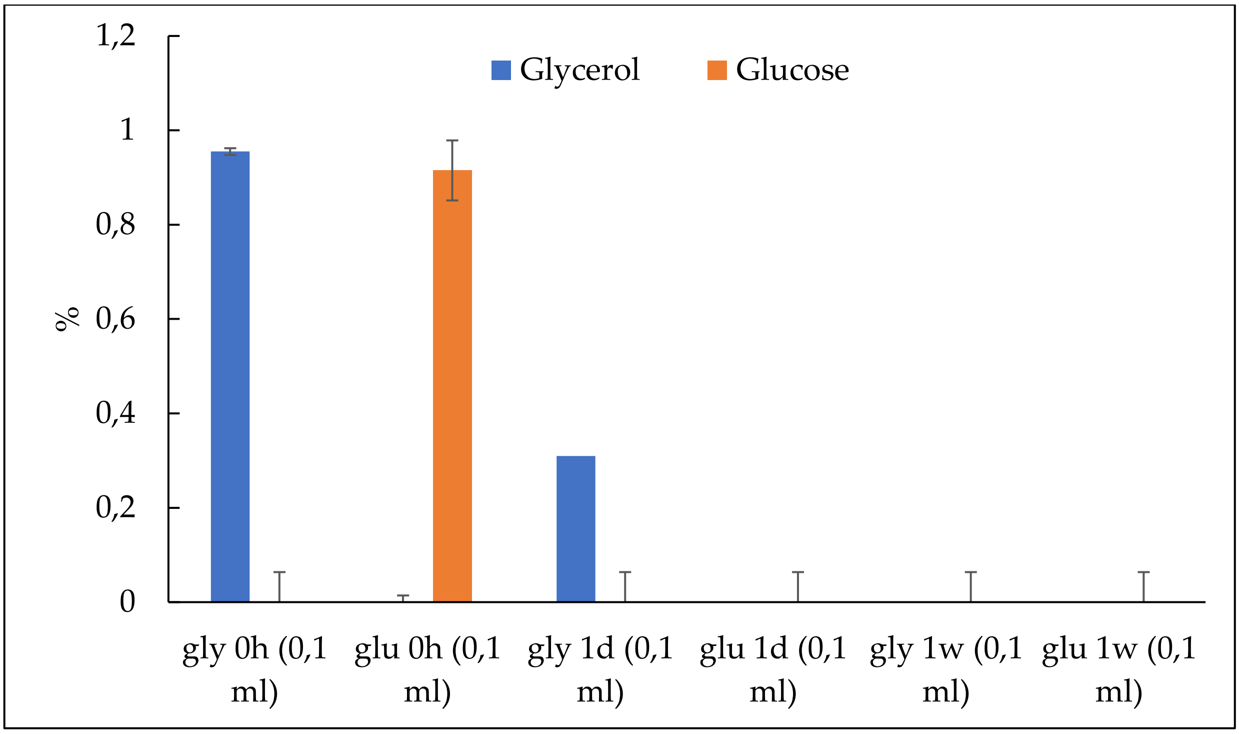

Figure S2. The level of growth substrates (glycerol and glucose) in the cultural medium upon the long-term cultivation of *Endomyces magnusii*. **Footnotes**: gly- 0h, glu- 0h; the initial level in the cultural medium; gly 1d, glu 1d; the 24 -hour growth stage; gly 1w, glu 1w the one-week growth stage.

Table S2. Semi-quantitative analysis of identified metabolites

Class	Compounds	MF	Approximate content in dry biomass, %	
			**min**	**max**
Carbohydrates	Glucose	862	0.8	4.3
	Galactose	811	0	0.10
	Lyxose	786**	0	0.08
	Maltose	828	0	0.002
	Ribose	808	0.001	0.2
	Stachyose	821	0	0.08
	Trehalose	813	0.0001	0.001
Deoxy Sugars	2-Deoxyglucose	791	0.0002	0.15
Carbohydrate lactones	Galactono-1,4-lactone	744**	0	0.18
	Gluconic acid lactone	804	0.001	0.07
Carbohydrate acids	Galacturonic acid	740	0	0.4
Phospho sugars	Fructose phosphate	816	0	0.009
	Galactose phosphate	867	0	0.12
Polyols	Myo-inositol	871	0.1	1.6
	Arabitol	949	2.8	7.0
	Galactinol	855	0	0.12
	Glycerol	915	0.001	6.7
	Sorbitol	902	0.01	1.4
	Erythritol	849	0.04	0.7
	Xylitol	912	0.001	0.09
	Mannitol	800	1.4	3.8
	Ribo-Hexitol, 3-deoxy	873	0.002	0.08
	**Dulcitol**	834	0.001	0.1
Polyols phosphates	Glycerol-3-phosphate	936	0.02	0.8
Amino acids	Glycine	800		<0.001
	Leucine	838		<0.001
	Isoleucine	802		<0.001
	Phenylalanine	882		<0.001
	Serine	881		<0.001
	Threonine	816		<0.001
	Valine	822		<0.001
	Pyroglutamic acid	889		<0.001
Ribonucleosides	Adenosine	708**		<0.001
Pyrimidine nucleosides	Uridine	826	0.001	0.04
Fatty acids	2-Ketobutyric acid	743**	0.001	0.15
	3-Hydroxyisobutyric acid	907	0	0.1
	2-Ketoisovaleric acid	916	0	0.1
	Caprylic acid	771**	0.004	0.01
	Elaidic acid	795**	0	0.08
	Linoleic acid	943	0	0.5
	Palmitic acid	940	0.01	0.8
	Stearic acid	858	0	0.2
Fatty acid derivatives	Oleic acid amide	800	0	0.1
Monoacylglycerols	Palmitoylglycerol	943	0	0.2
	Linoleoylglycerol	911	0	0.2
Sterols	Ergosterol	805	0	0.11
	Dehydroergosterol	793**	0	0.08

Dicarboxylic acids	Fumaric acid<br>Malic acid	807<br>907	0<br>0	0.02<br>0.4
Tricarboxylic acids	Citric acid<br>Succinic acid	811<br>944	0<br>0	0.07<br>0.3
Mono carboxylic acid	Lactic acid<br>3-Hydroxypropionic acid<br>Methyl glyceric acid	866<br>855<br>820	0.001<br>0<br>0	0.08<br>0.08<br>0.002
Carboxylic esters	Labdanol	807	0	0.02
Glycosides, O-glycosides	Lamiide (Cyclopenta[c]pyran-4-carboxylic acid, 1-(beta-D-glucopyranosyloxy)-1,4a,5,6,7,7a-hexahydro-4a,6,7-trihydroxy-7-methyl-, methyl ester)<br>**N.i. glycoside***	800<br>766	 0.1	0.001<br>1.5
Phenols	Gallic acid<br>Benzoic acid<br>Anthiaergostan<br>Anthiaergostan-5,7,9,16,22-penten<br>Anthiaergostan-5,7,9,22-tetraen-3-ol	869<br>772**<br>762**<br>741**<br>760**	0<br>0<br>0<br>0<br>0	<0.001<br>0.01<br>0.01<br>0.01<br>0.01
Inorganic acids	Phosphoric acid<br>Methyl-phosphoric acid	903<br>865	0.01<br>0.1	0.04<br>0.8

*** N.i. - not identified**

** Identification was performed using standards from the library by the resource center "Development of Molecular and Cellular Technologies" of St. Petersburg State University and the library of the Laboratory of Analytical Phytochemistry of the Botanical Institute of the Russian Academy of Sciences (St. Petersburg, Russia).
